# A Coupled Mechanobiological Model of Muscle Regeneration In Cerebral Palsy

**DOI:** 10.3389/fbioe.2021.689714

**Published:** 2021-08-27

**Authors:** Stephanie Khuu, Justin W. Fernandez, Geoffrey G. Handsfield

**Affiliations:** ^1^ Auckland Bioengineering Institute, The University of Auckland, Auckland, New Zealand; ^2^ Department of Engineering Science, The University of Auckland, Auckland, New Zealand

**Keywords:** agent-based modeling, mechanobiology, finite element modeling, FEM, skeletal muscle, satellite cell

## Abstract

Cerebral palsy is a neuromusculoskeletal disorder associated with muscle weakness, altered muscle architecture, and progressive musculoskeletal symptoms that worsen with age. Pathological changes at the level of the whole muscle have been shown; however, it is unclear why this progression of muscle impairment occurs at the cellular level. The process of muscle regeneration is complex, and the interactions between cells in the muscle milieu should be considered in the context of cerebral palsy. In this work, we built a coupled mechanobiological model of muscle damage and regeneration to explore the process of muscle regeneration in typical and cerebral palsy conditions, and whether a reduced number of satellite cells in the cerebral palsy muscle environment could cause the muscle regeneration cycle to lead to progressive degeneration of muscle. The coupled model consisted of a finite element model of a muscle fiber bundle undergoing eccentric contraction, and an agent-based model of muscle regeneration incorporating satellite cells, inflammatory cells, muscle fibers, extracellular matrix, fibroblasts, and secreted cytokines. Our coupled model simulated damage from eccentric contraction followed by 28 days of regeneration within the muscle. We simulated cyclic damage and regeneration for both cerebral palsy and typically developing muscle milieus. Here we show the nonlinear effects of altered satellite cell numbers on muscle regeneration, where muscle repair is relatively insensitive to satellite cell concentration above a threshold, but relatively sensitive below that threshold. With the coupled model, we show that the fiber bundle geometry undergoes atrophy and fibrosis with too few satellite cells and excess extracellular matrix, representative of the progression of cerebral palsy in muscle. This work uses *in silico* modeling to demonstrate how muscle degeneration in cerebral palsy may arise from the process of cellular regeneration and a reduced number of satellite cells.

## Introduction

Cerebral palsy (CP) is a neuromusculoskeletal disorder arising from a static neural lesion but leading to musculoskeletal and gait impairments that can give rise to long-term degradation of musculature (Fridén and Lieber, 2003; Smith et al., 2011; Larkin-Kaiser et al., 2019). CP is the most common cause of physical disability in children and manifests as spasticity, contractures, poor control of muscles, and altered reflexes and posture (Graham et al., 2016). Depending on disease severity, muscles in individuals with CP are often smaller and weaker than typically developing (TD) counterparts; additionally, muscle size and strength decline over time (Elder et al., 2003; Handsfield et al., 2016; von Walden et al., 2017; Sahrmann et al., 2019). The macroscale changes to CP muscle are well-known; however, cellular level studies of muscle regeneration are only commonly performed for TD muscle, as opposed to CP muscle. Of the few recent studies performed on CP muscle, cellular level differences between CP and TD muscle have been found and include increased collagen deposition in the extracellular matrix (ECM) (Booth et al., 2001; Fridén and Lieber, 2003; De Bruin et al., 2014), decreased number of muscle stem cells (Smith et al., 2013), decreased stem cell activity (Domenighetti et al., 2018), and an increase in pro-inflammatory gene expression compared to TD muscle (Von Walden et al., 2018).

Skeletal muscle is a post-mitotic tissue capable of repair and regeneration. Muscle regeneration and repair may be triggered by different cues including trauma, muscle use and strain, and chronic degenerative diseases, which over time lead to tissue adaptation (Artrong et al., 1991; Järvinen et al., 2008; Chen et al., 2020). Typically, eccentric exercise stimulation of muscles attenuates age-related muscle loss and promotes myofiber hypertrophy (Chen et al., 2020). Stimuli such as eccentric lengthening exercises cause mechanical strains in the muscle that damage cell membranes and lead to a cascade of chemical signals and cellular responses. Following damage, the muscle fiber environment undergoes a tightly regulated adaptive repair process which is often categorized according to a series of four phases of regeneration: 1) damage in the form of membrane rupture, 2) acute inflammatory response from macrophages and neutrophils, which involves breakdown and clearance of necrotic tissue, 3) regeneration orchestrated by activation, proliferation, differentiation of myogenic precursor cells and fusion of myoblasts to the debrided region of the myofiber, and 4) repair and remodeling of the ECM by fibroblasts (Partridge, 2002; Mourkioti and Rosenthal, 2005; Chargé and Rudnicki, 2009; Novak et al., 2014; Laumonier and Menetrey, 2016). During the first step, the breakdown and necrosis of myofibers is triggered via the disruption of the sarcolemma and subsequent increase in permeability. Creatine kinase is released into the serum and is a common marker of post-mechanical stress or muscle degeneration. Loss of calcium homeostasis and calcium influx, due to damage of the sarcolemma, then drives tissue necrosis. The result of injury is focal or total autolysis of fibers (Chargé and Rudnicki, 2009). Step two is marked by degeneration that occurs within hours of damage with the activation of myeloid and secretory cells that predominantly release cytokines. Neutrophils invade injured muscle within 1–6 h and reach peak concentration 6–24 h post-injury. At 48 h post-injury, macrophages become the predominant inflammatory cell types at the injury site. Macrophages phagocytose cellular debris and activate myogenic cells, ready for the regeneration process (Novak et al., 2014). Their importance in skeletal muscle regeneration is due to their phagocytic and antigen-presenting roles (Tidball and Villalta, 2010). Arnold et al., 2007 postulate that phagocytosis of muscle cell debris induces a switch of pro-inflammatory macrophages toward an anti-inflammatory phenotype, releasing TGF-β. This also suggests that inflammatory macrophages stimulate myogenic proliferation while anti-inflammatory macrophages exhibit differentiating activity.

The damaged environment activates quiescent satellite cells (SCs) and fibroblasts that remodel the affected tissue. SCs are a mononucleated, progenitor cell population pivotal to physiological muscle repair and regeneration. In an uninjured state, SCs sit between the plasma membrane of the muscle fiber and the basal lamina. SC content of muscle differs between age groups and activity levels. Increased SC density is observed at the neuromuscular junction and adjacent to capillaries. This suggests the muscle environment created by and surrounding these structures may attract SCs or regulate the distribution of the SC pool. The regulation of SC density is also observed down to the single myofiber level and during regeneration, activation of SCs is not restricted to the site of injury on the myofibre. Mechanical stimulation through endurance and resistance exercise can also accelerate the turnover of ECM components in skeletal muscle. The ECM is primarily composed of collagens, laminins, fibronectin and proteoglycans. Fibroblasts synthesize and assemble most of the ECM in skeletal muscle, while other components are responsible for degradation of the ECM (Lu et al., 2011). Collagen synthesis is increased post-exercise and transcriptional analysis shows increased encoding of collagen types I, III and IV post-endurance training (Grzelkowska-Kowalczyk, 2016). A loss of balance in terms of fibroblast secretion of ECM and clearance of collagen may result in perturbed muscle regeneration and fibrosis.

The muscle regeneration process builds new healthy muscle under optimal conditions and can be organized and considered as a four step process (Chargé and Rudnicki, 2009). It should be noted that perturbation of this process may lead to fatty infiltration and fibrotic tissue (Joe et al., 2010; Uezumi et al., 2010; Wang et al., 2015). While regeneration in skeletal muscle occurs at the cellular level, degeneration of CP muscle over time leads to both cellular and observable macroscale changes (Graham et al., 2016). In light of this, it bears considering whether the process of muscle regeneration may lead to degeneration in CP primarily as a result of changes to the cellular environment. The regeneration process is complex however, and exploration of this problem requires understanding interactions at multiple scales among multiple cellular agents.

Agent-based modeling (ABM) is a technique capable of probing complex adaptive systems from the bottom-up. In ABM, autonomous agents are situated in an environment with changeable relationships. ABM is well-suited for biology as bottom-up modeling enables cells to act and react to one another and to local stimuli without an *a priori* macroscale outcome. This is achieved through its representation of multiple levels of biological organization, capturing of intracellular dynamics between large populations of cells, and its ability to integrate cell-signaling events (An et al., 2009). Furthermore, both cellular and non-cellular components of an agent-based model can be programmed to perform biologically relevant behaviors such as proliferation, apoptosis and migration (Gorochowski, 2016). The macroscale behavior observed is then either a directed or an emergent effect of the local cellular actions and interactions; thus, ABM is a useful tool for capturing the complex, nonlinear, and multiscale nature of physiology (North et al., 2013; Wilensky and Rand, 2015), and is a promising approach to model the process of muscle regeneration. The ABM approach has previously been applied to studies of tumor formation, cardiac modeling, vascular remodeling, bone tissue regeneration, wound healing, signaling, and metabolic processes (Bailey et al., 2009; Flegg et al., 2015; Borgiani et al., 2017).

ABM has been used previously to explore muscle-related pathologies such as Duchenne muscular dystrophy (Virgilio et al., 2018) and disuse atrophy (Martin et al., 2015). Here, we develop an ABM that simulates typical muscle regeneration based on physiological properties and rules derived from literature. To simulate a muscle’s response to mechanical stimulus, we built a 3D finite element (FE) model of a muscle fiber bundle that underwent active eccentric contraction. The resultant areas of high strain and thus mechanical damage in the FE model serve as cues for remodeling in the agent-based model. This coupled model links mechanical stimulus and damage to its physiological response in skeletal muscle. We use this model to simulate the mechanobiological feedback loop of muscle repair over 3 months and investigate chronic regeneration and degeneration in TD and CP muscles. The purpose of this study was to simulate active eccentric contraction of muscle to obtain local strain data, then to seed the highest localized strain points into the agent-based model of muscle regeneration and evaluate the sensitivity of fibril recovery post-injury after altering SC concentration.

The pathological differences in muscle geometry and deformation that may lead to changes in the tissue microstructure can be observed by coupling ABM and FE modeling. Firstly, the FE analysis is used to determine areas of high strain and to localize tissue injury. These areas direct cell migration in the agent-based model as it is used to simulate a cycle of repair following injury. Secondly, following the repair time course, the resultant agent-based model geometry and cell counts can inform the reconstruction of the FE model geometry, thereby providing progressive morphological data. Lastly, as the two models provide repeated feedback, the effect of pathological muscle morphology on chronic injury and regeneration can be observed and compared to typical muscle regeneration and muscle morphology. By coupling FEM with ABM in an iterative fashion, the biological processes of tissue adaptation can be explored over time. This coupled model investigates how impairment in the muscle regeneration process can influence pathological muscle degeneration in CP. There is a need to better understand this process in individuals with CP over time.

## Methods

**FIGURE 1:**
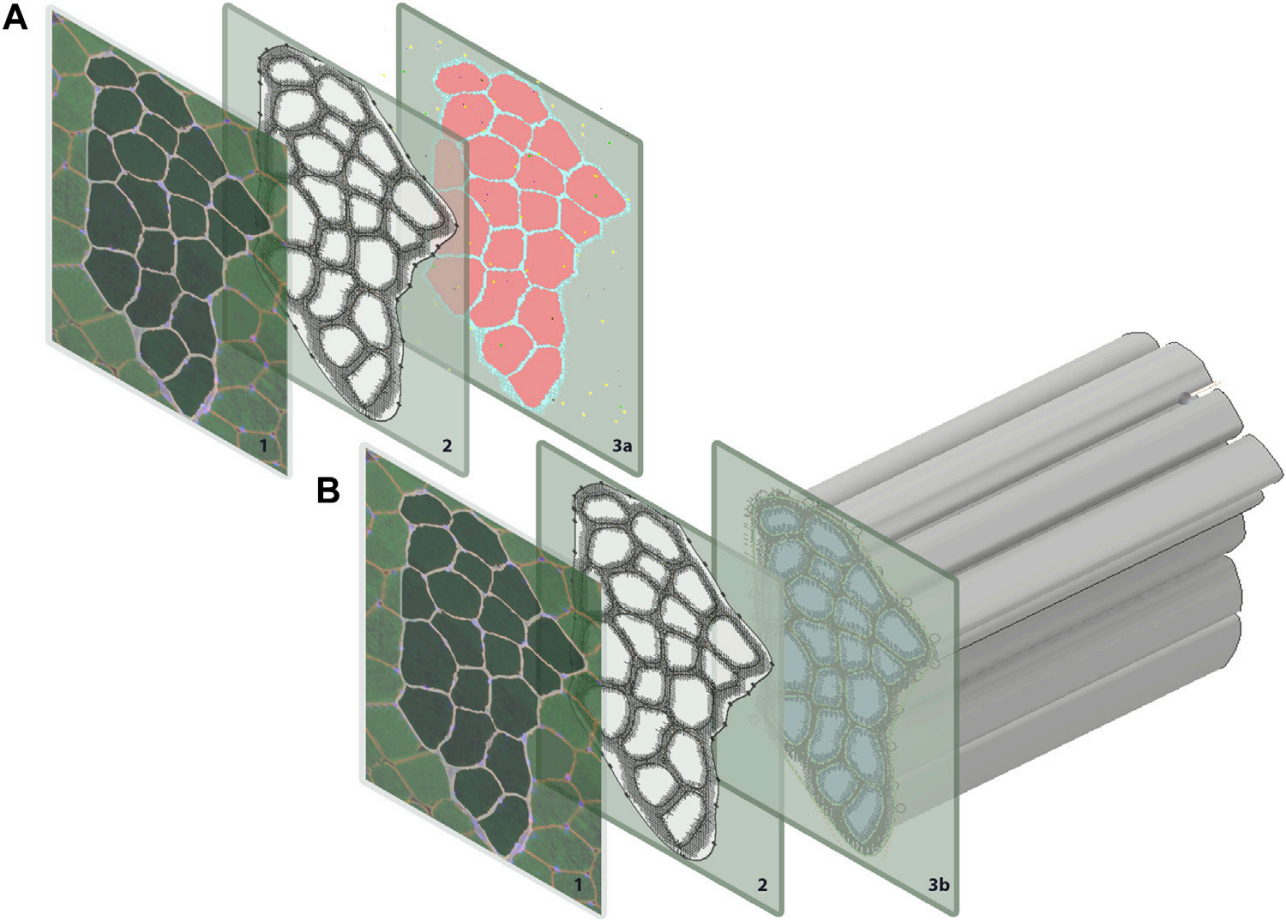
Geometry for both the agent-based models and FE models were generated from a single histological section. The coordinates for each pixel were extracted in MATLAB and used to recreate **(A)** pixels on ABM grid, and **(B)** to extrude muscle fibers for the FE models.

This study coupled an agent-based model and FE model of a human skeletal muscle fiber bundle to simulate muscle regeneration in response to eccentric contraction in CP and TD muscle. Initial geometry for all models was built from a single human muscle fiber cross-section obtained from the literature (Mackey et al., 2017), where this group undertook antibody staining of muscle biopsies from four young healthy male human participants. The vastus lateralis is a frequently used muscle in biopsy studies since it is a large, easily accessed muscle (Baczynska et al., 2016). In this case, we used a histological image from human vastus lateralis as a representative of fiber bundle architecture for human lower limb muscle. The histological image of muscle fiber bundle cross-section was segmented to distinguish 20 muscle fibers from the extracellular matrix (ECM) (Figure 1) using the Statistics and Machine Learning Toolbox™ in MATLAB (The MathWorks, Inc., Natick, MA). The 20 muscle fibers form a fiber bundle with each fiber surrounded by ECM and allows the study of cell-cell and cell-ECM interactions. Briefly, color-based K-means clustering was used to distinguish ECM from fibers, labeling every pixel in the image with a cluster index (1 or 0). The following sections discuss the agent-based model and FE model construction separately.

### Agent-Based Modeling

**FIGURE 2:**
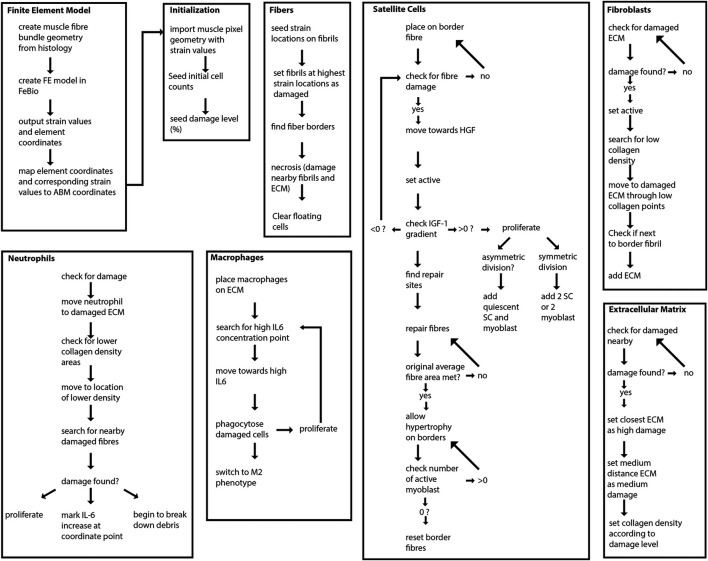
Overview of agent-based model steps. FE model resultant strains are mapped from each element center coordinate to the corresponding pixel coordinates of fibrils in the agent-based model. Fibers, SCs, neutrophils, macrophages, fibroblasts and ECM components work to regenerate damaged tissue.

The agent-based model was implemented in Repast Simphony (North et al., 2013), a Java-based modeling platform. Pixel values of the initial geometry were loaded onto a grid at the corresponding coordinate points in Repast Simphony (Figure 1A). The agent-based model contains 20 muscle fibers and represents a cross-sectional slice thickness of 50 µm. The ABM rules were developed based on literature descriptions of physiological interactions (see subsections below) (Figure 2). The model was built to simulate the progression of events during muscle regeneration known to take place over 4 weeks following injury (Laumonier and Menetrey, 2016).

Cell populations modeled as agents in the agent-based model include muscle fibers, macrophages and neutrophils, SCs, fibroblasts, and ECM components. Extracellular guidance cues influenced the behavior of agents. These extracellular guidance cues comprised the cytokines and growth factors IGF-1, TGF-ß, HGF, IL-6 and TNF-α (Eqs. 1–5). Cytokine levels were based on cell-type specific secretions or generalized cell secretions. The agent-based model simulated muscle regeneration over 28 days following 10% damage to muscle fibrils. Initial agent-based model geometry consisted of 11719 2D grid elements. The model simulated 28 days of regeneration with each tick representing 1 hour of cell activity. Each pixel represents 6.45 µm^2^ of tissue. Each simulation was run 150 times, unless otherwise stated, and simulation results are shown as mean ± standard deviation (SD).

Two ABM environments were developed to represent the muscle milieu in (1) a healthy TD skeletal muscle and (2) a CP skeletal muscle. Typical SC concentration in young adult males and children is reported as ∼0.10 SC per fiber for 10 µm slice thickness (Verdijk et al., 2014; Snijders et al., 2015; Mackey et al., 2017). A decrease in SC concentration ∼60% has been found in muscle biopsies from children with CP compared to typical adolescents (Smith et al., 2013). Therefore, SC density for 10 µm slice thickness was 0.10 SC per fiber for the healthy skeletal muscle and 0.04 SC per fiber for the CP muscle, representing a 60% decrease. For 50 µm slice thickness, the equation for SC counts was $SC\;density\;for\;10µm\times number\;of\;fibers\times \frac{slice\;thickness}{10}$. Initial SC counts were set to 0.1 × 20 × 5 = 10 for the TD muscle and 0.04 × 20 × 5 = 4 for CP muscle. We additionally conducted a sensitivity analysis to assess the impact of variation in SC count on the resulting fibril count over time.

#### Fibers and Extracellular Matrix

The creation of fiber and ECM geometry has been described above. Each agent was initialized to a coordinate point on a 2D grid based on the segmentation results. Damage was assigned to fiber and ECM regions in the agent-based model based on regions of high strain in the FE model (described in detail below). Following eccentric contractions, regions of strain above a certain threshold in muscle indicate the locations of injury (Best et al., 1995; Garrett, 1996). In the agent-based model, fibril agents with the highest 10% of strain values from FE modeling were regarded as damaged in the agent-based model. Neighbor fiber and ECM agents were also set to damaged, where neighbors were defined by searching the von Neumann neighborhoods of damaged fiber agents. This extended area of damage simulated necrotic tissue (Laumonier and Menetrey, 2016). Damaged ECM had collagen density set to a lower value and signaled for fibroblasts to initiate the repair process (Schoenrock et al., 2018). When average fibril count per fiber was repaired to pre-injury levels, fiber borders were re-formed and additional fibrils could be added, consistent with the “ghost fiber” phenomenon previously observed (Webster et al., 2016); at this point, hypertrophy could occur if any of the remaining circulating SCs were still active.

#### Neutrophils

For initial time steps of the agent-based model, neutrophils were distributed randomly on ECM grid points. Once damage had occurred, neutrophils searched their neighborhoods for damaged ECM and moved towards these points by updating their location to points where collagen density had declined since the previous time step. Neutrophils peak within 24 h (Smith et al., 2008). When neutrophils encountered damaged ECM or fibers, they proliferated, released IL-6 (Xue et al., 2015), and marked objects as needing repair. Neutrophils then broke down nearby damaged objects.

#### Macrophages

At initial time points, macrophages were localized to ECM. Once damage had occurred in the model, macrophages searched a Moore neighborhood for the highest IL-6 concentration and moved towards the corresponding grid location. Once damage was located, the angle of movement was computed, and the macrophages moved in this angular direction towards the damage. This method simulates the behavior of chemical factors that attract macrophages (Serrano et al., 2008;Muñoz‐Cánoves et al., 2013). When a macrophage encounters a damaged fiber, the fiber is set to “needs repair” and is eligible for phagocytosis (Tidball, 1995; Oishi and Manabe., 2018). Phagocytosis allows for macrophage proliferation and increases in the levels of TNF-α present due to the proliferation of pro-inflammatory M1 macrophages in the model (Ostrowski et al., 1998; Xue et al., 2015; Wynn and Vannella, 2016). After clearing damage, M1 macrophages switch their phenotype to anti-inflammatory M2 macrophages which release TGF-β (Delaney et al., 2017; Dort et al., 2019).

#### Satellite Cells

In our simulations, SCs are seeded according to physiological values of approximately 0.10 SCs per fiber per 10 µm thick section (Verdijk et al., 2014; Snijders et al., 2015; Mackey et al., 2017). During initialization, SCs are randomly assigned to border fibrils to represent their location between the sarcolemma and basement membrane of muscle fibers. The activation of quiescent SCs requires the presence of hepatocyte growth factor (HGF) (O’Reilly et al., 2008). SC division can take place after 18 h for each agent (Zammit et al., 2002). The proliferation of active SCs then occurs to mimic the transit-amplifying cells present during repair (Hsu et al., 2014). This process took place in a timeframe when the gradient of IGF-1 was greater than zero (Zanou and Gailly, 2013). Both symmetric and asymmetric division occurred. Symmetric division resulted in two satellite cells or two myoblasts, while asymmetric division produced one quiescent and one active satellite cell (Kuang et al., 2008). The chance of division decreased 20% after each of the first three divisions for a single SC, before decreasing by 40% at the fourth division (Siegel et al., 2011). Active SCs searched Moore neighborhoods for fibers that need repair and placed a myoblast at these locations.

#### Fibroblasts

Fibroblast levels were seeded according to Mackey et al. (2017). Fibroblasts became activated myofibroblasts in the presence of a positive TGF-β gradient (Ismaeel et al., 2019). Myofibroblasts searched the area for empty cells that neighbored ECM components. Myofibroblasts competed with myoblasts to regenerate tissue by depositing collagen near damaged ECM edges. When there were no SCs present in the muscle, fibroblasts deposited collagen in any remaining spaces.

#### Secreted Factors

Levels of secreted factors IGF-1 (Martin et al., 2015), HGF (Leuning et al., 2018), IL-6, TNF-α (Kim et al., 2011; Martin et al., 2015; Xue et al., 2015) and TGF-β (Vignola et al., 1996) per hour were represented by the following equations:

$$\frac{dIGF}{dt}=2(8.8\;e^{-5}\;AM)$$ (1)

$$\frac{dTNF}{dt}=3.21e^{-12}+5.8e^{-12}\;N+\;1.25e^{-9}\;PM+4.9e^{-18}\;Fb$$ (2)

$$\frac{dTGF}{dt}=8.75e^{-3}\;AM$$ (3)

$$\frac{dHGF}{dt}=1.49e^{-7}\;DE\;$$ (4)

$$\frac{dIL6}{dt}=2.91e^{-12}+1.25e^{-12}N+1.25e^{-12}PM$$ (5)

where AM was the number of anti-inflammatory macrophages, N was the number of neutrophils, PM was the number of pro-inflammatory macrophages, Fb was the number of fibroblasts, and DE was the number of damaged ECM components.

### Finite Element Modeling

The initial FE model was built from the same cross-sectional geometry that informed the initial agent-based model’s creation. Each subsequent FE model was built from the endpoint agent-based model geometry resulting from each ABM simulation. For the initial model, coordinate points from the segmented histological image were imported to Inventor^®^ (Autodesk., San Rafael, CA). The coordinates were then connected, and the muscle fiber bundle cross-section was extruded to represent 1 cm of muscle (Figure 1B). FE simulations were conducted in FEBio (Maas et al., 2012) and included muscle fibers and ECM. Muscle fibers were modeled with superposed active and passive stress, to simulate muscle activation in the fiber direction, where Cauchy stress is given by:

$$\sigma =\sigma_{p}+\sigma_{a}$$

Muscle fiber stress, $T_{a}$, was modeled using the time-varying elastance model in FEBio:

$$T_{a}=T_{max}\frac{Ca_{0}^{2}}{Ca_{0}^{2}+ECa_{50}^{2}}C\left(t\right)$$

$$ECa_{50}=T_{max}\frac{{\left(Ca_{0}\right)}_{max}}{\sqrt{\exp\left[B\left(l-l_{0}\right)\right]-1}}$$

Where tension of maximum isometric contraction $T_{max}=135.7kPa$, peak intracellular calcium concentration ${\left(Ca_{0}\right)}_{max}=4.35\mu M$, $B=4.75\mu m^{-1}$, l is the sarcomere length, and the length at which there is no active sarcomere tension is $l_{0}=1.58\mu m$. ECM and the passive component of the muscle fibres were modeled as nearly incompressible, hyperelastic materials, based on the following strain energy function for biological soft tissues (Weiss et al., 1996):

$$\Psi =F_{1}\left({\tilde{I}}_{1},{\tilde{I}}_{2}\right)+F_{2}\left(\tilde{\lambda }\right)+\frac{K}{2}{\left[\ln\left(J\right)\right]}^{2}$$

where Ψ is the strain energy functional, expressed uncoupled as the superposition of the ground substance Mooney-Rivlin response, $F_{1}$, the response of the fiber family, $F_{2}$, and the dilatational response where K is the bulk modulus and J is the Jacobian of the deformation tensor. The Mooney-Rivlin response is defined as:

$$F_{1}\left({\tilde{I}}_{1},{\tilde{I}}_{2}\right)=c_{1}\left({\tilde{I}}_{1}-3\right)+c_{2}\left({\tilde{I}}_{2}-3\right)$$

where ${\tilde{I}}_{1}$ and ${\tilde{I}}_{2}$ are the first and second invariant of the deviatoric right Cauchy-Green deformation tensor, $c_{1}$ and $c_{2}$ are material parameters. The fiber response is

F2(λ˜)={0,                                                             λ˜≤1 c3(e−c4(Ei(c4λ˜)−Ei(c4))−ln λ˜),  1≤λ˜≤λmc5(λ˜−1)+c6ln λ˜,                                 λ˜≥ λm

where Ei denotes the exponential integral function, defined for real non-zero values below:

$$Ei\left(x\right)=\;\overset{x}{\underset{-\infty }{\int }}\frac{e^{t}}{t}dt$$

$\tilde{\text{λ}}$ is deviatoric fiber stretch; and $c_{3}$, $c_{4}$, $\;c_{5\;}$and $c_{6}$represent material constants of the constitutive relations. Parameters are provided in Table 1. Fiber stress response is defined as:

$$\tilde{\text{λ}}\frac{\partial {\text{F}}_{2}}{\partial \tilde{\text{λ}}}=\{\begin{array}{c} 0,\;\;\;\;\;\;\;\;\;\;\;\;\;\;\;\;\;\;\;\;\;\;\;\;\;\;\;\;\;\;\;\;\;\;\;\;\;\;\;\;\;\;\;\;\;\;\;\;\;\;\;\;\;\tilde{\text{λ}}\leq 1\;\\ c_{3}(e^{c_{4}(\text{λ}˜-1)}-1),\;\;\;\;\;\;\;\;\;\;\;\;\;\;\;\;\;\;\;\;\;\;\;\;\;\;\;1\leq \tilde{\text{λ}}\leq {\text{λ}}_{m}\\ c_{5}\tilde{\text{λ}}+c_{6},\;\;\;\;\;\;\;\;\;\;\;\;\;\;\;\;\;\;\;\;\;\;\;\;\;\;\;\;\;\;\;\;\;\;\;\;\;\;\;\tilde{\text{ λ}}\geq \;{\text{λ}}_{m} \end{array}$$

**TABLE 1:** Material properties for ECM and fibers in the FE models of the muscle fiber bundle. Properties were unchanged across all iterations.

Properties	ECM	Fiber
material model	trans iso Mooney Rivlin	trans iso Mooney Rivlin
density	1	1
$c_{1}$	0.5	15
$c_{2}$	0	0
$c_{3}$	2	2
$c_{4}$	60	60
$c_{5}$	600	600
K	1,000	1,000

**FIGURE 3:**
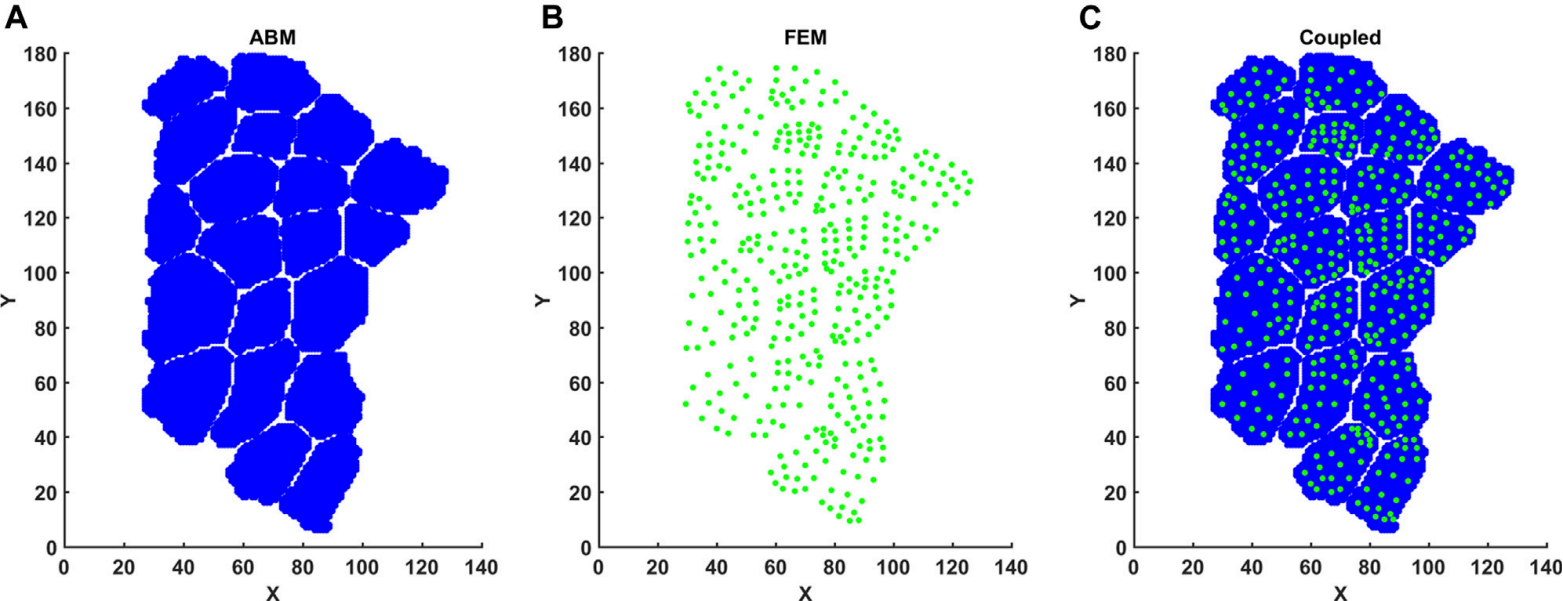
Mapping FE element centers into ABM coordinate space. **(A)** FE element centers were calculated based on nodal points. **(B)** ABM pixels were imported with associated X, Y coordinates. **(C)** Element center coordinates and associated strain per element were then registered to ABM coordinates.

Note that we used one set of constitutive parameters for both CP and TD FE models. Eccentric contraction was simulated by imposing a fiber stretch of 30% with one end of the muscle fiber bundle held fixed. Fibers underwent maximum voluntary active contraction during eccentric load. Activation parameters are given below (Table 2). At the end of FE simulations, element centers were calculated based on average coordinates for node points. Strain values were then matched with element centers before being mapped onto the 2D coordinate points of the agent-based model (Figure 3). Inhomogeneous strain values over the area of the fibers were parsed into the agent-based model.

**TABLE 2:** Active contraction properties.

**Properties**	**ECM**	**Fiber**
ascl	1	1
ca0	4.35	4.35
beta	4.75	4.75
I0	1.58	1.58
refl	2.04	2.04

### Coupled Model

**FIGURE 4:**
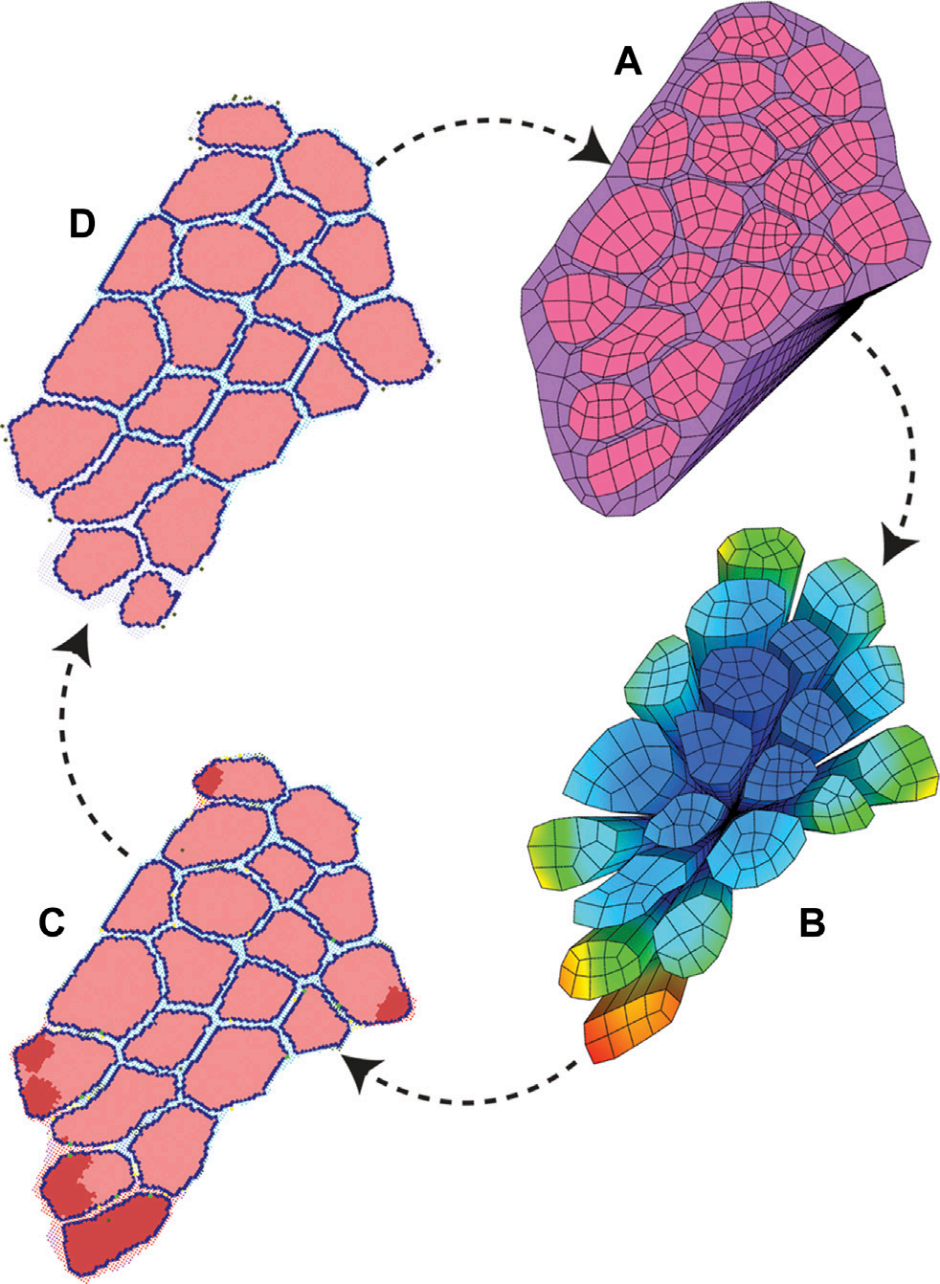
ABM and FE modeling coupling workflow. The initial pixel values and coordinates were used to build both starting ABM and FE modeling geometry. **(A)** FE simulations were run to model active eccentric contraction of a fiber bundle. **(B)** FE strain values were recorded with associated coordinate points. **(C)** Points of high strain were imported into the agent-based models as local damage. The agent-based models were run, and mean endpoint geometry **(D)** was used to generate a new FE geometry.

Coupling of these models was achieved by simulating eccentric contraction and damage via strain using the FE models, followed by simulation of regeneration using ABM, where the endpoint geometry was then used for a new FE model. The strain values from the FE model were mapped to coordinate points of the ABM and the highest 10% of strain values were used to mark fibrils at relevant locations as damaged in the ABM following initialization (Figure 2). Two agent-based models were used for two separate simulation series: one agent-based model representing CP muscle and another representing TD muscle. Each agent-based model in our framework simulated 28 days of regeneration. The two seeded initial ABM environments represented TD muscle with an initial SC count of 10, or CP muscle with an initial SC count of 4. These simulation environments were run separately, and results from each agent-based model were used to create separate endpoint geometries: CP post-regeneration vs TD post-regeneration. Each endpoint geometry was exported to generate new FE meshes for the coupled simulation’s next iteration (Figure 4). FE constitutive properties were kept consistent across iterations. After each FE simulation, strain values were exported and parsed to the agent-based model, where they were registered with the correct coordinates (Figure 3). We chose a representative ABM geometry output near the mean, which was used to create the next FE model. This process was repeated three times to simulate 3 months of muscle damage and regeneration. Three months is the time it takes to investigate three full cycles of regeneration following injury, without the added complexity of increased simulation time and physiological processes such as growth. Since constitutive properties remained unchanged—across iterations and between TD and CP models—this approach probed the role that SCs have in muscle regeneration and the potential for chronic degeneration in the case of impaired SC density.

## Results

**FIGURE 5:**
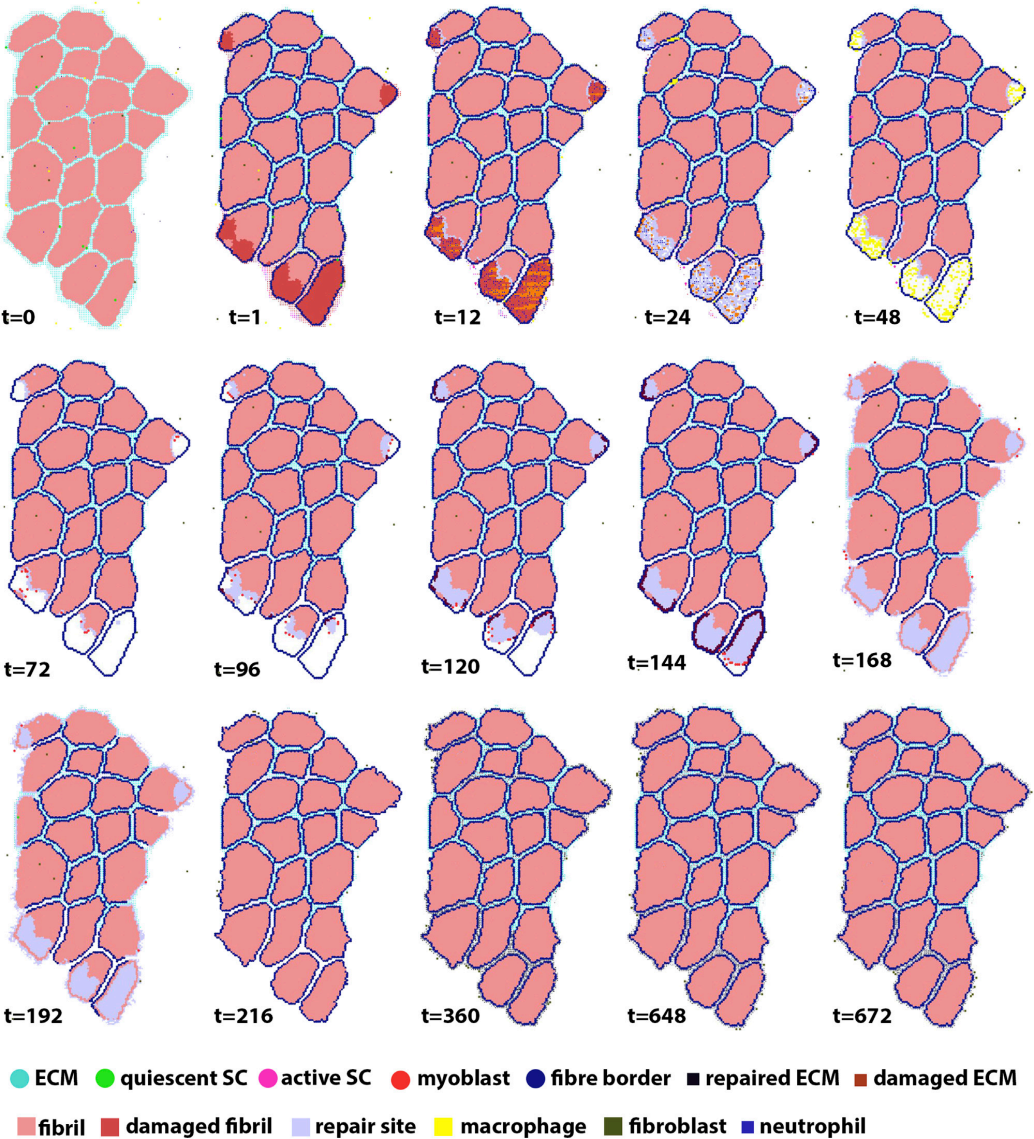
ABM simulation of muscle regeneration over time. At the first time point, regions of the muscle fiber bundle show damage. This damage signals for the mobilization of macrophages, neutrophils, satellite cells and fibroblasts, which interact in the muscle regeneration process. Note that the dominant change from *t* = 216 to *t* = 672 is ECM remodeling.

Each agent-based model simulated 672 h (28 days) of muscle regeneration. Figure 5 illustrates the progression of regeneration following injury as simulated by our agent-based model. At t = 1, 10% of fibers were damaged, and neutrophil invasion began and lasted 24 h. This was followed by macrophage invasion over the first 3 days. Once macrophages cleared the necrotic tissue, SCs began the repair process. Fiber repair was completed at 216 h post-injury; hypertrophy occurred in cases where the environment was suitable, i.e., when there were still active SCs in the environment after the damaged fibers were replaced and boundaries re-formed. In these cases, border fibrils were re-formed by recalculating the final fibrils that were next to ECM, to accommodate hypertrophy. In the last stages of repair, fibroblasts acted to fill the remaining gaps with ECM.

FE material parameters ($c_{1}$, $c_{3}$, $c_{4}$, $c_{5}$, K and density) were altered by ±10% to investigate changes in strain due to different input values for the material properties (Table 3). For both ECM and Fiber materials, a 10% increase in parameter $c_{5}$ decreased the maximum strain value by 7.07%; a 10% decrease in $c_{5}$ increased the maximum strain value by 7.29%. Percentage change in minimum strain was also altered by $c_{5}$ where a 10% increase in $c_{5}$ decreased the minimum strain by 2.16%, and a 10% decrease in $c_{5}$ increased the minimum strain by 2.17%. For parameters $c_{1}$, $c_{3}$, $c_{4}$, K and density, ± 10% variations in input values resulted in less than 1% change in maximum and minimum strain values.

**TABLE 3:** Sensitivity analysis of FE material model parameters for ECM and Fibers. Model values were varied by ±10% (input values) and percentage changes to the maximum and minimum strain in each FE simulation are shown.

**Material**	**Parameter**	**Model value**	**Input value**	**% Change maximum strain**	**% Change minimum strain**
**ECM**	c1	0.5	0.45	0.0490	0.1160
			0.55	−0.0473	−0.1029
	c3	2	2.2	0.0001	0.0000
			1.8	0.0001	0.0000
	c4	60	66	0.0001	0.0000
			54	0.0001	0.0000
	c5	600	660	−7.0741	−2.1556
			540	7.2902	2.1744
	k	1,000	1,100	-0.6683	-0.2091
			900	0.5573	0.1748
	density	1	1.1	0.0001	0.0000
			0.9	0.0001	0.0000
**Fiber**	c1	15	16.5	0.0490	0.1160
			13.5	−0.0473	−0.1029
	c3	2	2.2	0.0001	0.0000
			1.8	0.0001	0.0000
	c4	60	66	0.0001	0.0000
			54	0.0001	0.0000
	c5	600	660	−7.0741	−2.1556
			540	7.2902	2.1744
	k	1,000	1,100	−0.6683	−0.2091
			900	0.5573	0.1748
	density	1	1.1	0.0001	0.0000
			0.9	0.0001	0.0000

**FIGURE 6:**
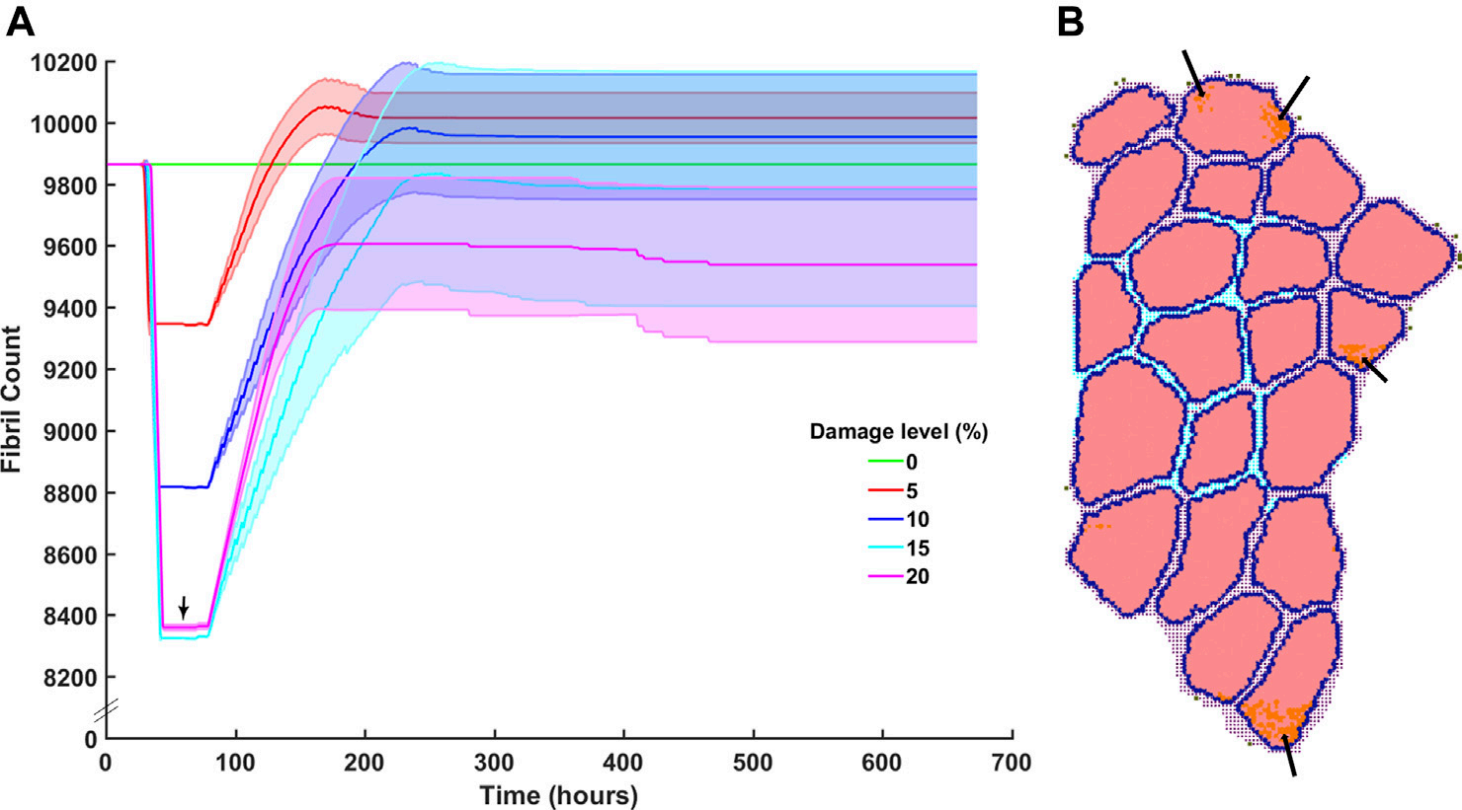
Fibril recovery post injury at varied damage levels of 0% fibrils to 20% with 5% steps. **(A)** Fibril recovery at 5 and 10% showed average end fibril count increase. At 15% clearance of damaged cells was effective and repair was close to recovery, however, at 20% damage, the clearance of damaged fibrils did not reach 20% (arrow) and therefore **(B)** inflammatory cells and damaged fibrils remain within the fibers.

Damage levels with additional necrosis were varied from 0 to 20% at 5% increments to explore the range in which efficient muscle regeneration can be simulated (Figure 6A). At 5 and 10% damage levels, the clearance of damaged fibrils was completed within 90 h post injury and recovery of original fibril count was achieved. 5% damage led to endpoint fibril count of 10,020 ± 80 (mean ± SD) and 10% led to endpoint fibril count of 9,954 ± 206 (mean ± SD). 15% on average was able to clear the damage but unable to reach original fibril levels (endpoint mean ± SD 9785 ± 385). With extensive damage of 20%, there was insufficient clearance of damaged fibrils (Figure 6B, arrows), and repair that occurred around those damaged fibrils did not reach the original count of 9,864 fibers.

**FIGURE 7:**
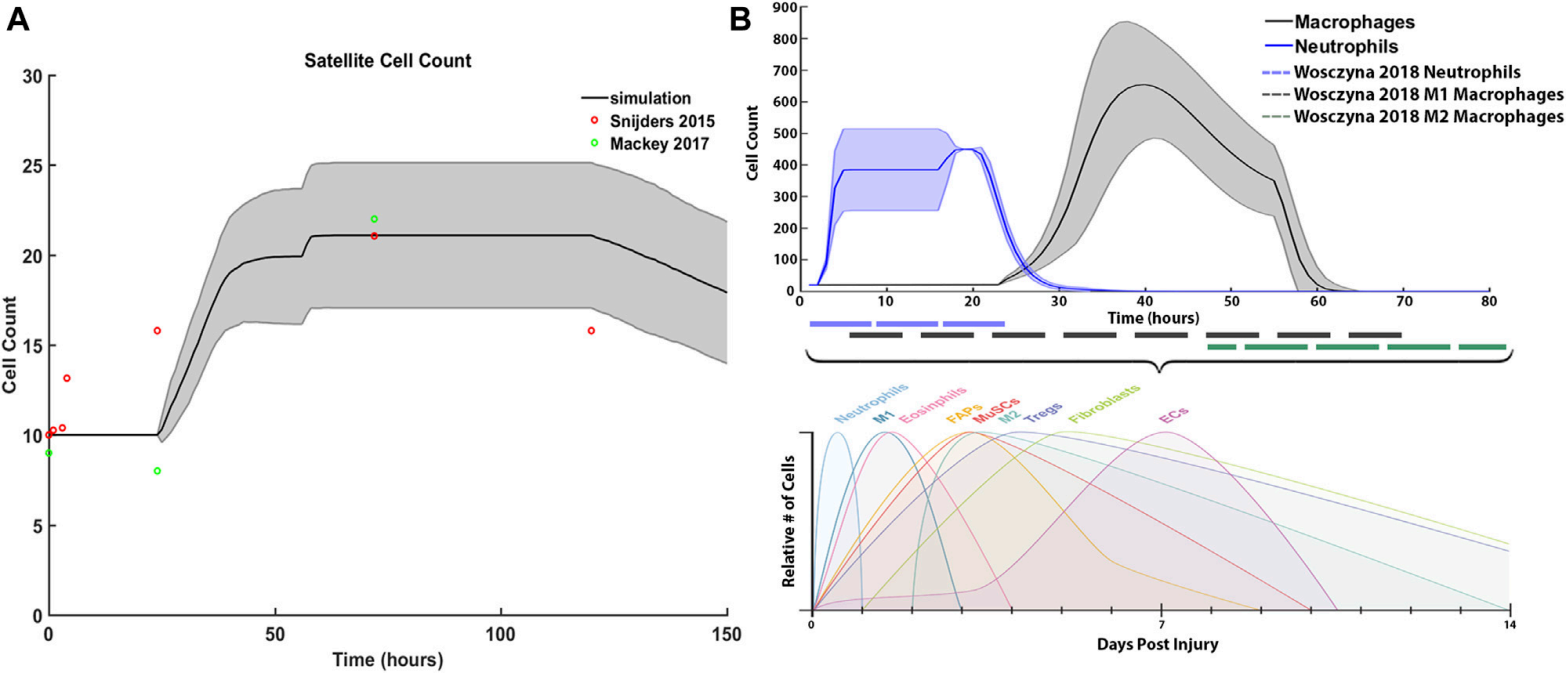
ABM simulated cell counts over time. **(A)** SC simulation results (mean ± SD) compared to Snijders et al. (Snijders et al., 2015) **(B)** Simulated inflammatory cell counts of neutrophil and macrophage (mean ± SD) over time (solid line) compared to the generalized time course of when neutrophil, M1 macrophage and M2 macrophage cell counts are above 20% from baseline (dashed line), adapted from (Wosczyna and Rando, 2018).

We compared our simulated SC with literature values from Snijders et al. (2015) and Mackey et al. (2017), and inflammatory cell counts with Wosczyna and Rando (2018) (Figure 7). For SCs, initial and average peak values at 72 h are comparable; however, SC proliferation did not occur at the same initial rate compared to the Snijders et al. Simulated proliferation stalled for the first 24 h and then rose sharply while literature data showed a gradual rise in SCs. Simulated SC counts started to decline at 120 h, which was similar to the literature (Figure 7A). Average neutrophil counts in the agent-based models peaked at 20 h under typical ABM conditions, while macrophages peaked around 40 h. The decrease in macrophage count in the ABM simulations occurred in sync with the generalized time course for M1 macrophages, as evidenced from Wosczyna and Rando (2018) (Figures 7B,C). However, simulated macrophage count did not perfectly replicate the time course for M2 macrophages. This is partially due to the nature of the ABM, where the macrophage count combined both M1 and M2 macrophages as one cell population that underwent a switch at the appropriate time.

**FIGURE 8:**
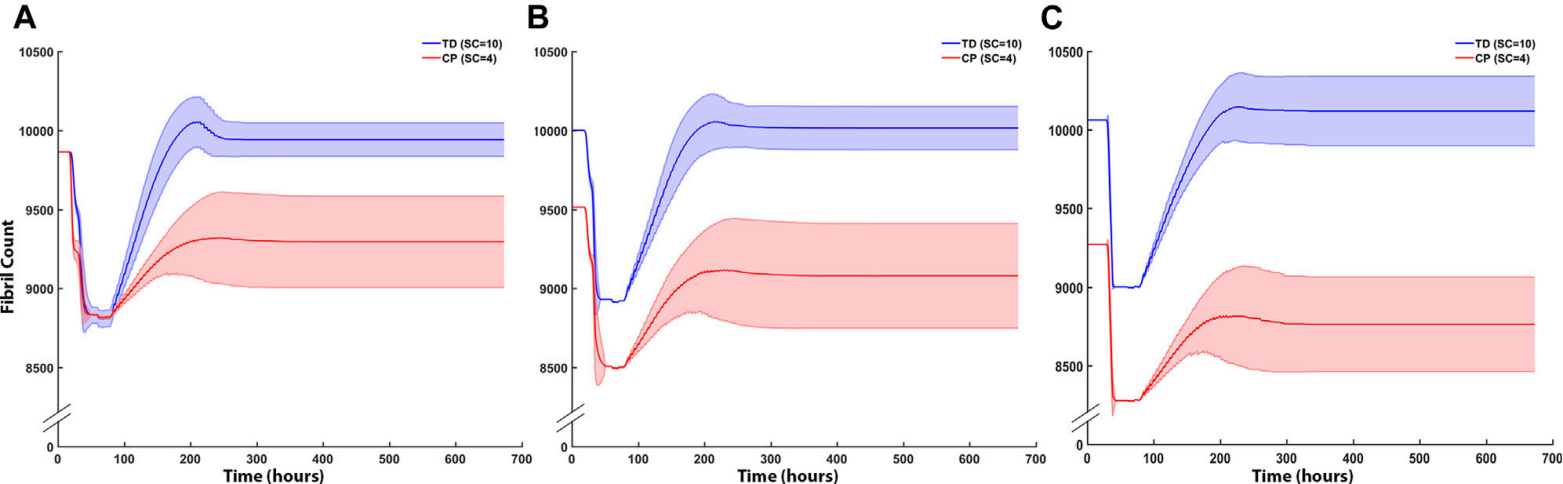
ABM cell counts over time (mean ± SD) seeded with CP (SC = 4) or TD (SC = 10) initial conditions. **(A)** Iteration one cell counts over the first 672 h, based on initial geometry. TD fibril count exceeded initial count whereas CP fibril recovery was impaired. **(B)** ABM cell counts (mean ± SD) using TD and CP iteration two geometry and strain values seeded with CP (SC = 4) or TD (SC = 10) conditions. TD fibril recovery continued to exceed initial counts and CP fibril recovery was further impaired. **(C)** Iteration three of the ABM cell counts. TD fibril count peaked above the original value during repair however stabilised to just below initial values by the end of the simulation. CP fibril recovery decreased to 8,763 ± 315 (mean ± SD).

Fibril counts over the three iterations demonstrate the divergent outcomes between the TD and CP muscle milieu (Figure 8). In the first iteration ABM simulation for both CP and TD environments, the initial geometries were identical, and the initial fibril count was 9,864 for both models (Figure 8A). Damage was cleared at approx. t = 50 h and regeneration began at t = 70 h when SCs were activated. In the TD scenario, recovery over initial fibril count occurred at t = 200 h and a 0.79% increase in fibril count above original count (9,942 ± 108) was observed. In the CP seeded agent-based models, recovery peaked at 200 h but the model was not able to recover to original fibril count (9,296 ± 289). In the second iteration ABM simulation (Figure 8B), TD and CP environments were seeded with 10,000 and 9,515 fibrils, respectively, due to altered geometry that resulted from the first iteration. For both scenarios, a 10% damage threshold was applied again. Over the 28 days of simulated regeneration, the TD environment repaired its fibril count to just above original levels (0.02%; fibril count = 10,002 ± 130). Over the same 28 days, the CP environment did not repair to original levels of fibrils and reached a final fibril count of 9,081 ± 331. The initial fibril counts for the third iteration (Figure 8C) of the ABM simulations were 10,060 (TD) and 9,272 (CP). For both cases, repair began at t = 78 h. TD fibril count reached a fibril count of 10,080 ± 260, which is above the initial fibril count for the third iteration. CP recovery was again unable to repair to original counts, completing its regeneration cycle at a fibril count of 8,763 ± 301.

**FIGURE 9:**
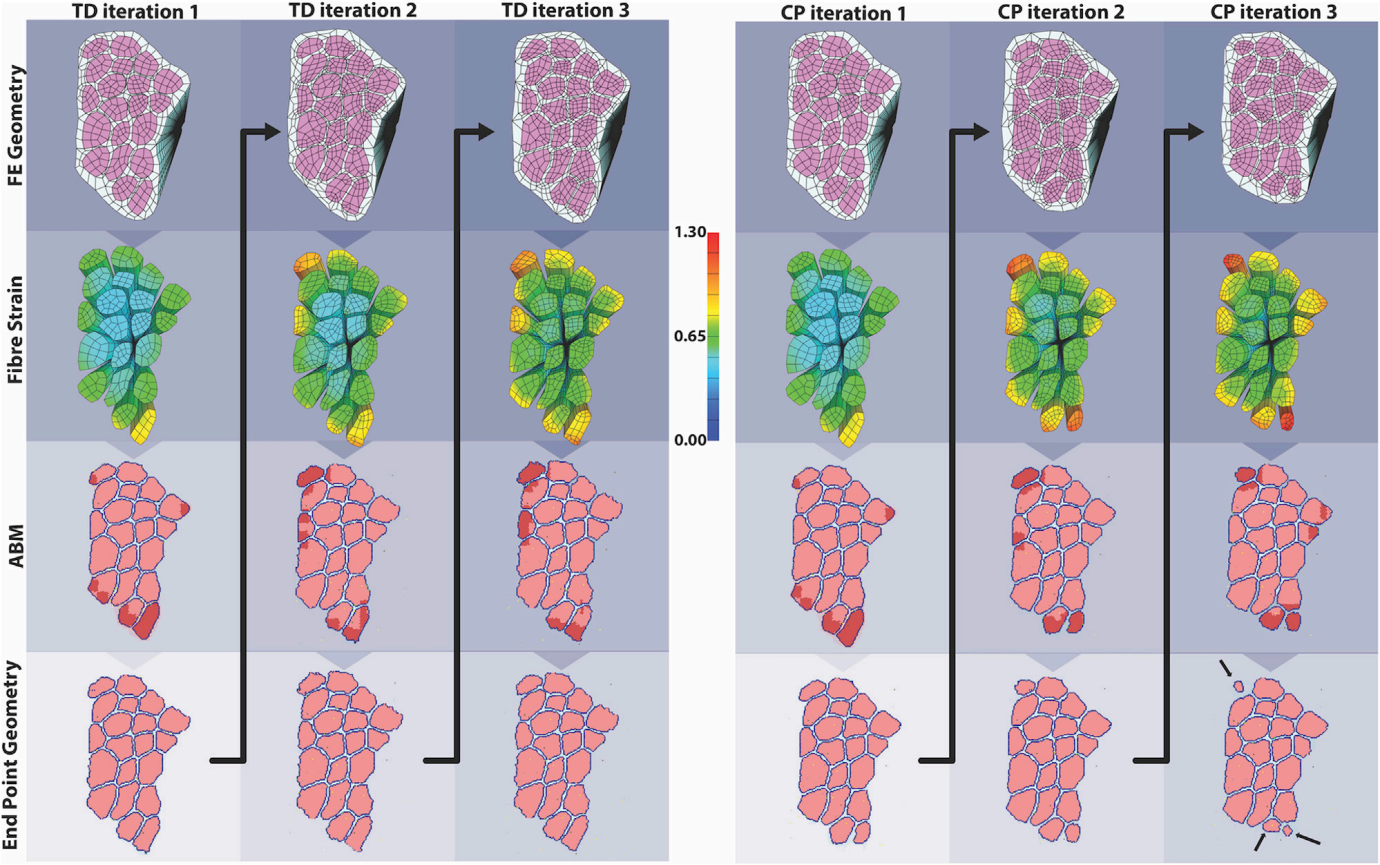
In the coupled ABM-FE modeling mechanobiological simulations, the initial geometry leads to two unique endpoint geometries, according to whether the agent-based models had a cellular milieu based on CP or TD muscle. Each of those endpoint geometries then leads to a new FE model geometry and simulation, where high strains differ based on the geometry from the previous step. Ultimately, divergent geometries emerge, reflecting the different CP vs TD muscle outcomes, where TD muscle regenerates fully each cycle and CP muscle fibers cyclically degenerate.

The coupled agent-based model and FE model simulated cyclic damage and regeneration in TD and CP muscle, which manifested as progressive degeneration in the CP model. When the FE model geometry simulated eccentric lengthening of muscle, the regions of highest strain occurred on outer fibers, particularly those located on the corners of the geometry. These regions were then assigned high strain and damage in the agent-based model. For the TD coupled model, i.e., the model in which the simulated muscle milieu represented TD muscle, the agent-based model repaired all of the damage and grew larger than the initial geometry with an increase in mean muscle fraction from 84.1 to 84.7% (*p*<<0.001), demonstrating emergent hypertrophy in these simulations. For the CP coupled model, i.e. the model in which the simulated muscle milieu represented CP muscle, the agent-based models were unable to repair the damage induced by the FE model (Figure 9) and the mean muscle fraction declined from 84.1 to 79.2% (*p*<<0.001). In the second TD iteration, the FE model displayed more widely distributed high strain values on outer fibers compared to the first iteration. In this second iteration of the TD model, the agent-based models again showed an increase in size of fibers affected by damage (muscle fraction = 85.2%, *p*<<0.001), again simulating emergent hypertrophy. In contrast, in the second iteration of the CP model, the highest strain in the FE model was concentrated on the smallest outer fibers, and in the ABM simulations, this damage was not entirely repaired, failing to restore the geometry to its original geometry and further decreasing muscle fraction to 77.3%. In the third iteration, fiber geometry showed a marginal increase in size in the TD model. The CP fibers continued to decrease in size in the third iteration, where muscle fraction fell to 79.6% and three fibers in particular demonstrated considerable atrophy (Figure 9 arrows). Average fiber cross sectional area increased to 3,251 µm^2^ for TD scenario, and decreased to 2,533 µm^2^ for CP, from original area of 3,182 µm^2^, over 3 months.

**FIGURE 10:**
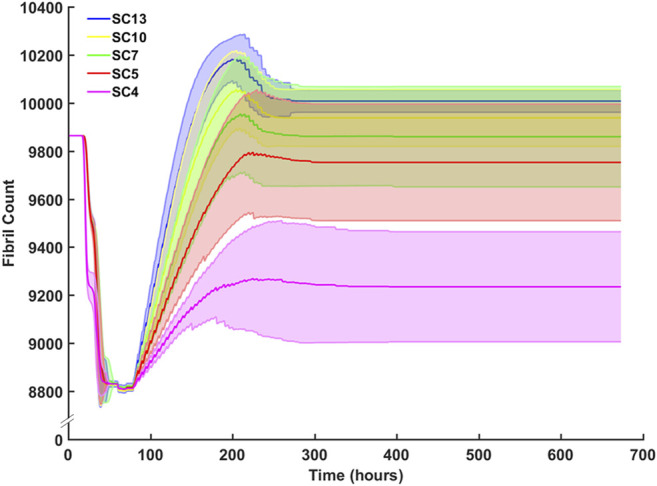
Sensitivity analysis for SC count and the effect on fibril count (mean ± SD) over simulation time course. SC count was set to 4 (CP), 5, 7, 10 (TD), and 13. Mean fibril count increased with increase in seeded SC count.

Sensitivity analysis for SC counts of 4 (CP), 5, 7, 10 (TD) and 13 were performed and evaluated with respect to fibril recovery (Figure 10). Mean fibril recovery increased with seeded SC count. SC counts of 4, five and seven had greater variance compared to SC counts of 10 and 13. Higher seeded SC count was inversely related to the number of hours required for recovery of initial fibril count. A 50% reduction of SCs reduced average fibril recovery count by only 110 fibrils while a 30% reduction in seeded SC count led to mere marginal decreases in endpoint fibril recovery. An SC count of 4, however, resulted in a more dramatic decrease of 523 fibrils compared to an SC count of 5.

**FIGURE 11:**
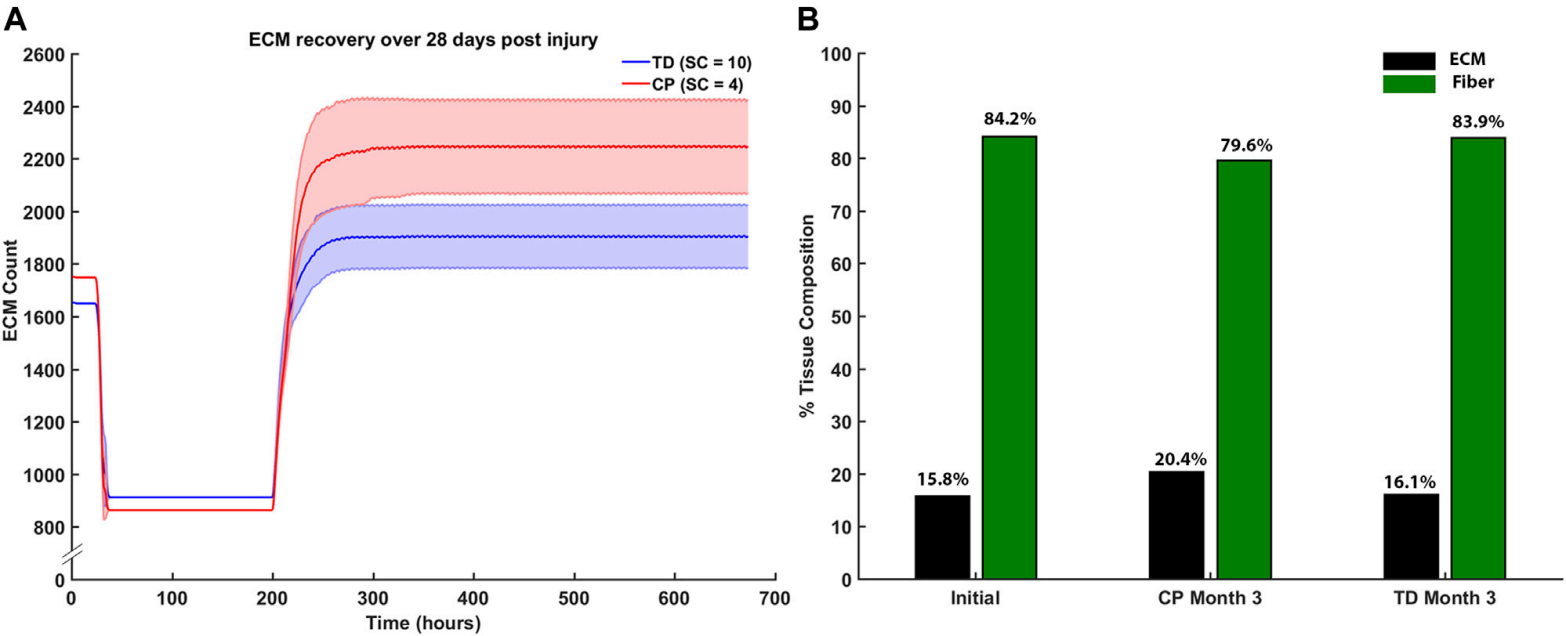
**(A)** ECM recovery over the third month 28 days post injury simulation. CP muscle environment (SC = 4) had higher end point ECM count of 2,240 ± 176 and the TD environment had a lower endpoint count of 1932 ± 139. **(B)** Tissue composition changed from initial simulations to the end of the third month. ECM percentage increased by 4.6% in the CP scenario and marginally increased by 0.3% in the TD scenario.

Endpoint ECM count was measured after the third month of regeneration for CP and TD environments (Figure 11A). The CP environment had an endpoint ECM count of 2,240 ± 176 and the TD environment had a lower endpoint count of 1,932 ± 139. Tissue composition was from the initial simulation was calculated and compared to both the third month averages for CP and TD simulations (Figure 11B). The third month endpoint CP ECM made up 20.4% of the tissue, increased from 15.8%. In comparison, the third month endpoint TD ECM increased marginally to 16.1% from initial. While the TD ECM fraction and muscle fractions were stable over 3 months, the CP muscle fraction decreased from 84.2% to 79.6%.

## Discussion

In this work, we coupled agent-based modeling with finite element modeling of a muscle fiber bundle to explore the interaction of muscle damage and regeneration in the context of altered satellite cells and supporting cells in the muscle fiber environment. We were particularly interested in whether this framework could demonstrate a progressive degeneration of the muscle fiber bundle consistent with the progression of cerebral palsy.

Our coupled model demonstrates the canonical process of muscle regeneration after several bouts of damage from eccentric contraction (Charge and Rudnicki, 2004; Toumi et al., 2006; Järvinen et al., 2008; Wang and Rudnicki, 2012; Novak and Koh, 2013). Over the course of 672 h, our simulations showed damage, inflammation, clearance of damaged tissue, repair of muscle tissue regions, and remodeling of the extracellular matrix. The regeneration model includes quiescence, activation, and proliferation of satellite cells; M1 and M2 macrophages and neutrophils; fibroblasts; muscle fibers and extracellular matrix agents; and secreted factors of TNF-α, TNF-β, IGF-1, and HGF. Each of these factors and agents interacts with one another in a bottom-up and nonlinear approach. The timeframe for repair in the typical scenario was 14–21 days, in line with active muscle regeneration studies where repair peaks at 2 weeks and subsequently declines (Ambrosio et al., 2009). Here, we imposed 10% damage at the initiation of each simulated iteration. It is possible that more extensive damage would have required a longer simulation time frame to repair fully; however, consistent in the literature are specific timeframes for different stages of repair. This suggests that the cellular response may scale in magnitude with the level of damage, rather than with the response timeframe. With this said, there are certainly practical limits on the extent of damage that can be repaired in skeletal muscle that is shown by incomplete clearance of damaged tissue in the 20% damage scenario. Prolonged presence of damaged fibers and resultant cellular debris due to insufficient inflammatory cell response has been shown to delay muscle regeneration (Summan et al., 2006). Our choice of 10% damage is thought to represent a reasonable level of mechanical damage that does not exceed skeletal muscle’s typical ability to mount the repair process.

Our models were consistent with literature reports of peak satellite cell concentrations and timing of inflammatory cell concentrations (Snijders et al., 2015; Wosczyna and Rando, 2018), and demonstrate other well-characterized phenomena observed in skeletal muscle regeneration such as ghost fibers (Webster et al., 2016) and fibrosis (Booth et al., 2001; Peterson et al., 2012; Kinney et al., 2017). The rules for the present model focus on post-injury cell counts during regeneration in healthy individuals after exercise-induced mechanical damage (McKay et al., 2010; Mackey and Kjaer, 2016; Kim and Lee, 2017; Nederveen et al., 2017). While this is a suitable model for exploring typical muscle injury and regeneration, this remains a largely unexplored area in the context of CP. Additionally, directional cues for non-cellular systemic agents such as cytokines have not been well-described in the literature. Lacking this information, the present models relied on cytokines that were concentrated at damage sites. Future models might consider using geometrical features to inform directional cues for the diffusion of signaling factors. For example, the inclusion of capillary location within the geometry could enhance the spatial localization of satellite cells and systemic cytokines within the model. However, at this stage, the functions of systemic and local signaling factors on muscle repair are yet to be fully explained (Chargé and Rudnicki, 2009); future experimental exploration and inclusion in computational models will be an exciting area in this field.

Sensitivity analysis demonstrated relative insensitivity of fibril count to satellite cell concentration between SC levels of 5 and 13; however, the level of five was such that, on average, simulations did not fully repair the damage induced in them and fibril count was lower at the end of the simulation. SC counts below five had drastically impaired ability to repair damage. The behavior of satellite cells in our model suggests a threshold of satellite cell concentration, above which is sufficient to repair 10% damage, and below which the damage repair falls off sharply. We performed additional sensitivity analyses on the constitutive parameters in our finite element model. While strain values were relatively insensitive to perturbations in most constitutive parameters, predictably we found a larger dependence of strain on c5, the along-fiber modulus. Overall, since the role of the finite element model was to identify regions of high strain within the geometry, the exact values of constitutive parameters are not thought to be of central importance to this goal. In this coupled modeling framework, we used strain as the mechanical parameter associated with damage in the muscle fiber bundle. Several prior studies reinforce this association (Lieber and Friden, 1993; Best et al., 1995; Lieber and Fridén, 1999). Mechanobiology is an active field, however, and it is unclear how other parameters such as strain rate, repeated loading, shear, and stress may contribute to the signaling and processes of muscle regeneration. Future modeling and experimental work may explore this area in finer detail.

Our framework involving FE modeling and ABM demonstrated a progressive degeneration of muscle fibers in the simulations resembling cerebral palsy muscle. The cerebral palsy muscle here was denoted by a reduced satellite cell concentration. Here, for simplicity and direct comparison, we did not alter material properties between the CP and TD FE models, and we did not change material properties over the course of simulations. It is arguable that CP muscle would have different constitutive properties compared to TD, such as stiffer muscle, or that changes over time to the muscle could well be captured with changes to material properties. The mode of injury used in this model was also limited to eccentric lengthening. Concentric exercise can result in hypertrophy; however, eccentric exercise is more efficient at eliciting hypertrophy and muscle growth (Schoenfeld et al., 2017). In any case, given the same form and extent of injury, whether by concentric or eccentric loading, the repair mechanism would likely remain the same.

In this model, we chose eccentric contraction as a standard model that causes fiber damage (and initiates the muscle repair process) and is physiologically relevant. From that stimulus, we simulate and observe the regeneration process. Damage frequency from eccentric strain may be caused more than once per month, however this time course was used to explore the entire four step process of muscle regeneration that is commonly referred to in the literature. Additionally, it is known that strain is localised during eccentric contraction and subsequent injury occurs in the areas of highest strain (above a threshold) (Lieber and Friden, 1993; Best et al., 1995; Garrett, 1996). In a test case, random damage was seeded across the same agent-based model (Supplementary Figure S1). This model was unable to clear cellular debris at 10% damage level and prevented ECM remodeling. This suggests that muscle repair in response to eccentric contraction vs inflammatory myopathies require different responses, the latter of which is beyond the scope of this study.

The purpose of the current model was to demonstrate if the process of muscle regeneration could lead to degeneration with reduced SC numbers. Under this proposition alone, our model illustrated this phenomenon, and the endpoint geometries observed here are similar to those seen in the literature for cerebral palsy muscle cross-sections (Marbini et al., 2002; Foran et al., 2005; De Bruin et al., 2014), namely reduced muscle fiber cross-sections and increased area fraction of ECM. To make a more robust geometry selection for the subsequent iterations, an average pixel geometry could be generated in the future versions of this model. Over 3 months, the muscle fraction declined in cerebral palsy simulations by 4.6%. In these simulations, the change in muscle geometry due to decrease in fiber size of damaged fibers represents atrophy at the fiber level. Under the framework that we have presented here, this degenerated muscle architecture characteristic of CP emerges from the same initial muscle geometry as in the TD model. The only difference was the muscle milieu, i.e. number of satellite cells, and thus the regeneration process of the muscle after injury.

## Conclusion

Coupled modeling is a powerful tool in its ability to connect tissue and organ level behaviors to simple cellular interactions. This model demonstrated growth over time in a TD muscle environment that experienced strain similar to that which would occur from active eccentric lengthening. In simulations of CP muscle environment, the same strains led to gradual degradation of size and shape of muscle fibers over time. Overall, this work suggests a plausible connection consistent with the physiological mechanisms that are observed in the clinical manifestation of cerebral palsy.

## Data Availability

The raw data supporting the conclusions of this article will be made available by the authors, without undue reservation.

## References

[B1] Ambrosio F. Kadi F. Lexell J. Fitzgerald G. K. Boninger M. L. Huard J. (2009). The Effect of Muscle Loading on Skeletal Muscle Regenerative Potential. Am. J. Phys. Med. Rehabil. 88, 145–155. 10.1097/PHM.0b013e3181951fc5 19169178 PMC4872620

[B2] An G. Mi Q. Dutta-moscato J. (2009). Agent-based Models in Translational Systems Biology 10.1002/wsbm.045 PMC3640333 20835989

[B3] Armstrong R. B. Warren G. L. Warren J. A. (1991). Mechanisms of Exercise-Induced Muscle Fibre Injury. Sports Med. 12, 184–207. 10.2165/00007256-199112030-00004 1784873

[B4] Arnold L. Henry A. Poron F. Baba-Amer Y. van Rooijen N. Plonquet A. (2007). Inflammatory Monocytes Recruited after Skeletal Muscle Injury Switch into Antiinflammatory Macrophages to Support Myogenesis. J. Exp. Med. 204, 1057–1069. 10.1084/jem.20070075 17485518 PMC2118577

[B5] Baczynska A. M. Shaw S. Roberts H. C. Cooper C. Aihie Sayer A. Patel H. P. (2016). Human Vastus Lateralis Skeletal Muscle Biopsy Using the Weil-Blakesley Conchotome. JoVE 2016, 53075. 10.3791/53075 PMC4828222 26967381

[B6] Bailey A. M. Lawrence M. B. Shang H. Katz A. J. Peirce S. M. (2009). Agent-based Model of Therapeutic Adipose-Derived Stromal Cell Trafficking during Ischemia Predicts Ability to Roll on P-Selectin. Plos Comput. Biol. 5, e1000294. 10.1371/journal.pcbi.1000294 19247427 PMC2636895

[B7] Best T. M. McElhaney J. H. Garrett W. E. Myers B. S. (1995). Axial Strain Measurements in Skeletal Muscle at Various Strain Rates. J. Biomech. Eng. 117, 262–265. 10.1115/1.2794179 8618377

[B8] Booth C. M. Cortina-Borja M. J. F. Theologis T. N. (2001). Collagen Accumulation in Muscles of Children with Cerebral Palsy and Correlation with Severity of Spasticity. Dev. Med. Child. Neurol. 43, 314–320. 10.1111/j.1469-8749.2001.tb00211.x 11368484

[B9] Borgiani E. Duda G. N. Checa S. (2017). Multiscale Modeling of Bone Healing: Toward a Systems Biology Approach. Front. Physiol. 8, 287. 10.3389/fphys.2017.00287 28533757 PMC5420595

[B10] Chargé S. B. P. Rudnicki M. A. (2004). Cellular and Molecular Regulation of Muscle Regeneration. Physiol. Rev. 84, 209–238. 10.1152/physrev.00019.2003 14715915

[B11] Chargé S. B. P. Rudnicki M. A. (2004). Cellular and Molecular Regulation of Muscle Regeneration. Physiol. Rev. 84, 209–238. 10.1152/physrev.00019.2003 14715915

[B12] Chen W. Datzkiw D. Rudnicki M. A. (2020). Satellite Cells in Ageing: Use it or Lose it. Open Biol. 10, 200048. 10.1098/rsob.200048 32428419 PMC7276531

[B13] De Bruin M. Smeulders M. J. Kreulen M. Huijing P. A. Jaspers R. T. (2014). Intramuscular Connective Tissue Differences in Spastic and Control Muscle: A Mechanical and Histological Study. PLoS One 9, e101038. 10.1371/journal.pone.0101038 24977410 PMC4076209

[B14] Delaney K. Kasprzycka P. Ciemerych M. A. Zimowska M. (2017). The Role of TGF-Β1 during Skeletal Muscle Regeneration. Cell Biol. Int. 41, 706–715. 10.1002/cbin.10725 28035727

[B15] Domenighetti A. A. Mathewson M. A. Pichika R. Sibley L. A. Zhao L. Chambers H. G. (2018). Loss of Myogenic Potential and Fusion Capacity of Muscle Stem Cells Isolated from Contractured Muscle in Children with Cerebral Palsy. Am. J. Physiology-Cell Physiol. 315, C247–C257. 10.1152/ajpcell.00351.2017 PMC6139501 29694232

[B16] Dort J. Fabre P. Molina T. Dumont N. A. (2019). Macrophages Are Key Regulators of Stem Cells during Skeletal Muscle Regeneration and Diseases. Stem Cell Int. 2019, 1–20. 10.1155/2019/4761427 PMC6664695 31396285

[B17] Elder G. C. B. BSc G. S. Pt K. C. MSc D. W. Marshall A. Leahey L. (2003). Contributing Factors to Muscle Weakness in Children with Cerebral Palsy. Dev. Med. Child. Neurol. 45, 542–550. 10.1111/j.1469-8749.2003.tb00954.x 12882533

[B18] Flegg J. A. Menon S. N. Maini P. K. McElwain D. L. S. (2015). On the Mathematical Modeling of Wound Healing Angiogenesis in Skin as a Reaction-Transport Process. Front. Physiol. 6, 1–17. 10.3389/fphys.2015.00262 26483695 PMC4588694

[B19] Foran J. R. Steinman S. Barash I. Chambers H. G. Lieber R. L. (2005). Structural and Mechanical Alterations in Spastic Skeletal Muscle. Dev. Med. Child. Neurol. 47, 713–717. 10.1017/S0012162205001465 16174321

[B20] Fridén J. Lieber R. L. (2003). Spastic Muscle Cells Are Shorter and Stiffer Than normal Cells. Muscle Nerve 27, 157–164. 10.1002/mus.10247 12548522

[B21] Garrett W. E. (1996). Muscle Strain Injuries. 8947416

[B22] Gorochowski T. E. (2016). Agent-based Modelling in Synthetic Biology. Essays Biochem. 60, 325–336. 10.1042/EBC20160037 27903820 PMC5264505

[B23] Graham H. K. Rosenbaum P. Paneth N. Dan B. Lin J.-P. Damiano D. L. (2016). Cerebral Palsy. Nat. Rev. Dis. Primers 2, 19–20. 10.1038/nrdp.2015.82 PMC9619297 27188686

[B24] Grzelkowska-Kowalczyk K. (2016). “The Importance of Extracellular Matrix in Skeletal Muscle Development and Function,” in Composition And Function Of the Extracellular Matrix In the Human Body, 3–24. 10.5772/62230

[B25] Handsfield G. G. Meyer C. H. Abel M. F. Blemker S. S. (2016). Heterogeneity of Muscle Sizes in the Lower Limbs of Children with Cerebral Palsy. Muscle Nerve 53, 933–945. 10.1002/mus.24972 26565390

[B26] Hsu Y.-C. Li L. Fuchs E. (2014). Transit-amplifying Cells Orchestrate Stem Cell Activity and Tissue Regeneration. Cell 157, 935–949. 10.1016/j.cell.2014.02.057 24813615 PMC4041217

[B27] Ismaeel A. Kim J.-S. Kirk J. S. Smith R. S. Bohannon W. T. Koutakis P. (2019). Role of Transforming Growth Factor-β in Skeletal Muscle Fibrosis: A Review. Ijms 20, 2446. 10.3390/ijms20102446 PMC6566291 31108916

[B28] Järvinen T. A. Kääriäinen M. Äärimaa V. Järvinen M. Kalimo H. (2008). “Skeletal Muscle Repair after Exercise-Induced Injury,” in In Skeletal Muscle Repair And Regeneration. Editors Schiaffino S. Partridge T. (Springer), 217–242. 10.1007/978-1-4020-6768-6

[B29] Joe A. W. B. Yi L. Natarajan A. Le Grand F. So L. Wang J. (2010). Muscle Injury Activates Resident Fibro/adipogenic Progenitors that Facilitate Myogenesis. Nat. Cel Biol. 12, 153–163. 10.1038/ncb2015 PMC4580288 20081841

[B30] Kim H. Kim H.-S. Youn J.-C. Shin E.-C. Park S. (2011). Serum Cytokine Profiles in Healthy Young and Elderly Population Assessed Using Multiplexed Bead-Based Immunoassays. J. Translational Med. 9, 113. 10.1186/1479-5876-9-113 PMC3146842 21774806

[B31] Kim J. Lee J. (2017). Role of Transforming Growth Factor-β in Muscle Damage and Regeneration: Focused on Eccentric Muscle Contraction. J. Exerc. Rehabil. 13, 621–626. 10.12965/jer.1735072.536 29326892 PMC5747195

[B32] Kinney M. C. Dayanidhi S. Dykstra P. B. McCarthy J. J. Peterson C. A. Lieber R. L. (2017). Reduced Skeletal Muscle Satellite Cell Number Alters Muscle Morphology after Chronic Stretch but Allows Limited Serial Sarcomere Addition. Muscle Nerve 55, 384–392. 10.1002/mus.25227 27343167 PMC5183525

[B33] Kuang S. Gillespie M. A. Rudnicki M. A. (2008). Niche Regulation of Muscle Satellite Cell Self-Renewal and Differentiation. Cell Stem Cell 2, 22–31. 10.1016/j.stem.2007.12.012 18371418

[B34] Larkin-Kaiser K. A. Howard J. J. Leonard T. Joumaa V. Gauthier L. Logan K. (2019). Relationship of Muscle Morphology to Hip Displacement in Cerebral Palsy: A Pilot Study Investigating Changes Intrinsic to the Sarcomere. J. Orthop. Surg. Res. 14, 1–9. 10.1186/s13018-019-1239-1 31227002 PMC6588916

[B35] Laumonier T. Menetrey J. (2016). Muscle Injuries and Strategies for Improving Their Repair. J. Exp. Ortop 3, 1–9. 10.1186/s40634-016-0051-7 PMC4958098 27447481

[B36] Leuning D. G. Beijer N. R. M. du Fossé N. A. Vermeulen S. Lievers E. Van Kooten C. (2018). The Cytokine Secretion Profile of Mesenchymal Stromal Cells Is Determined by Surface Structure of the Microenvironment. Sci. Rep. 8. 10.1038/s41598-018-25700-5 PMC5956003 29769543

[B37] Lieber R. L. Fridén J. (1999). Mechanisms of Muscle Injury after Eccentric Contraction. J. Sci. Med. Sport 2, 253–265. 10.1016/S1440-2440(99)80177-7 10668762

[B38] Lieber R. L. Friden J. (1993). Muscle Damage Is Not a Function of Muscle Force but Active Muscle Strain. J. Appl. Physiol. 74, 520–526. 10.1152/jappl.1993.74.2.520 8458765

[B39] Lu P. Takai K. Weaver V. M. Werb Z. (2011). Extracellular Matrix Degradation and Remodeling in Development and Disease. Cold Spring Harbor Perspect. Biol. 3, a005058. 10.1101/cshperspect.a005058 PMC3225943 21917992

[B40] Maas S. A. Ellis B. J. Ateshian G. A. Weiss J. A. (2012). FEBio: Finite Elements for Biomechanics. J. Biomech. Eng. 134, 011005. 10.1115/1.4005694 22482660 PMC3705975

[B41] Mackey A. L. Kjaer M. (2017). Connective Tissue Regeneration in Skeletal Muscle after Eccentric Contraction-Induced Injury. J. Appl. Physiol. 122, 533–540. 10.1152/japplphysiol.00577.2016 27562842

[B42] Mackey A. L. Magnan M. Chazaud B. Kjaer M. (2017). Human Skeletal Muscle Fibroblasts Stimulate *In Vitro* Myogenesis and *In Vivo* Muscle Regeneration. J. Physiol. 595, 5115–5127. 10.1113/JP273997 28369879 PMC5538230

[B43] Marbini A. Ferrari A. Cioni G. Bellanova M. F. Fusco C. Gemignani F. (2002). Immunohistochemical Study of Muscle Biopsy in Children with Cerebral Palsy. 10.1016/S0387-7604(01)00394-1 11891093

[B44] Martin K. S. Blemker S. S. Peirce S. M. (2015). Agent-based Computational Model Investigates Muscle-specific Responses to Disuse-Induced Atrophy. J. Appl. Physiol. 118, 1299–1309. 10.1152/japplphysiol.01150.2014 25722379 PMC4436981

[B45] McKay B. R. Toth K. G. Tarnopolsky M. A. Parise G. (2010). Satellite Cell Number and Cell Cycle Kinetics in Response to Acute Myotrauma in Humans: Immunohistochemistryversusflow Cytometry. J. Physiol. 588, 3307–3320. 10.1113/jphysiol.2010.190876 20624792 PMC2976024

[B46] Mourkioti F. Rosenthal N. (2005). IGF-1, Inflammation and Stem Cells: Interactions during Muscle Regeneration. Trends Immunol. 26, 535–542. 10.1016/j.it.2005.08.002 16109502

[B47] Muñoz‐Cánoves P. Scheele C. Pedersen B. K. Serrano A. L. (2013). Interleukin‐6 Myokine Signaling in Skeletal Muscle: a Double‐edged Sword? FEBS J. 280, 4131–4148. 10.1111/febs.12338 23663276 PMC4163639

[B48] Nederveen J. P. Snijders T. Joanisse S. Wavell C. G. Mitchell C. J. Johnston L. M. (2017). Altered Muscle Satellite Cell Activation Following 16 Wk of Resistance Training in Young Men. Am. J. Physiology-Regulatory, Integr. Comp. Physiol. 312, R85–R92. 10.1152/ajpregu.00221.2016 PMC5283938 27834290

[B49] North M. J. Collier N. T. Ozik J. Tatara E. R. Macal C. M. Bragen M. (2013). Complex Adaptive Systems Modeling with Repast Simphony. Complex Adapt. Syst. Model. 1, 1–26. 10.1186/2194-3206-1-3

[B50] Novak M. L. Koh T. J. (2013). Phenotypic Transitions of Macrophages Orchestrate Tissue Repair. Am. J. Pathol. 183, 1352–1363. 10.1016/j.ajpath.2013.06.034 24091222 PMC3969506

[B51] Novak M. L. Weinheimer-Haus E. M. Koh T. J. (2014). Macrophage Activation and Skeletal Muscle Healing Following Traumatic Injury. J. Pathol. 232, 344–355. 10.1002/path.4301 24255005 PMC4019602

[B52] O'Reilly C. McKay B. Phillips S. Tarnopolsky M. Parise G. (2008). Hepatocyte Growth Factor (HGF) and the Satellite Cell Response Following Muscle Lengthening Contractions in Humans. Muscle Nerve 38, 1434–1442. 10.1002/mus.21146 18816607

[B53] Oishi Y. Manabe I., (2018). Macrophages in Inflammation, Repair and Regeneration. 10.1093/intimm/dxy054 30165385

[B54] Ostrowski K. Rohde T. Zacho M. Asp S. Pedersen B. K. (1998). Evidence that Interleukin-6 Is Produced in Human Skeletal Muscle during Prolonged Running. J. Physiol. 508, 949–953. 10.1111/j.1469-7793.1998.949bp.x 9518745 PMC2230908

[B55] Partridge T. A. (2002). Cells that Participate in Regeneration of Skeletal Muscle. Gene Ther. 9, 752–753. 10.1038/sj.gt.3301764 12032703

[B56] Peterson M. D. Gordon P. M. Hurvitz E. A. Burant C. F. (2012). Secondary Muscle Pathology and Metabolic Dysregulation in Adults with Cerebral Palsy. Am. J. Physiology-Endocrinology Metab. 303, E1085–E1093. 10.1152/ajpendo.00338.2012 PMC3492860 22912367

[B57] Sahrmann A. S. Stott N. S. Besier T. F. Fernandez J. W. Handsfield G. G. (2019). Soleus Muscle Weakness in Cerebral Palsy: Muscle Architecture Revealed with Diffusion Tensor Imaging. PLoS One 14, e0205944–16. 10.1371/journal.pone.0205944 30802250 PMC6388915

[B58] Schoenfeld B. J. Ogborn D. I. Vigotsky A. D. Franchi M. V. Krieger J. W. (2017). Hypertrophic Effects of Concentric vs. Eccentric Muscle Actions: A Systematic Review and Meta-Analysis. J. Strength Cond. Res. 31, 2599–2608. 10.1519/JSC.0000000000001983 28486337

[B59] Schoenrock B. Zander V. Dern S. Limper U. Mulder E. Veraksitš A. (2018). Bed Rest, Exercise Countermeasure and Reconditioning Effects on the Human Resting Muscle Tone System. Front. Physiol. 9, 810. 10.3389/fphys.2018.00810 30018567 PMC6037768

[B60] Serrano A. L. Baeza-Raja B. Perdiguero E. Jardí M. Muñoz-Cánoves P. (2008). Interleukin-6 Is an Essential Regulator of Satellite Cell-Mediated Skeletal Muscle Hypertrophy. Cel Metab. 7, 33–44. 10.1016/j.cmet.2007.11.011 18177723

[B61] Siegel A. L. Kuhlmann P. K. Cornelison D. (2011). Muscle Satellite Cell Proliferation and Association: New Insights from Myofiber Time-Lapse Imaging. Skeletal Muscle 1, 7. 10.1186/2044-5040-1-7 21798086 PMC3157006

[B62] Smith C. Kruger M. J. Smith R. M. Myburgh K. H. (2008). The Inflammatory Response to Skeletal Muscle Injury. Sports Med. 38, 947–969. 10.2165/00007256-200838110-00005 18937524

[B63] Smith L. R. Chambers H. G. Lieber R. L. (2013). Reduced Satellite Cell Population May lead to Contractures in Children with Cerebral Palsy. Dev. Med. Child. Neurol. 55, 264–270. 10.1111/dmcn.12027 23210987 PMC4054943

[B64] Smith L. R. Lee K. S. Ward S. R. Chambers H. G. Lieber R. L. (2011). Hamstring Contractures in Children with Spastic Cerebral Palsy Result from a Stiffer Extracellular Matrix and Increasedin Vivosarcomere Length. J. Physiol. 589, 2625–2639. 10.1113/jphysiol.2010.203364 21486759 PMC3115830

[B65] Snijders T. Nederveen J. P. McKay B. R. Joanisse S. Verdijk L. B. van Loon L. J. C. (2015). Satellite Cells in Human Skeletal Muscle Plasticity. Front. Physiol. 6, 283. 10.3389/fphys.2015.00283 26557092 PMC4617172

[B66] Summan M. Warren G. L. Mercer R. R. Chapman R. Hulderman T. Van Rooijen N. (2006). Macrophages and Skeletal Muscle Regeneration: A Clodronate-Containing Liposome Depletion Study. Am. J. Physiology-Regulatory, Integr. Comp. Physiol. 290, R1488–R1495. 10.1152/ajpregu.00465.2005 16424086

[B67] Tidball J. G. (1995). Inflammatory Cell Response to Acute Muscle Injury. Med. Sci. Sports Exerc. 27, 1022–1032. 10.1249/00005768-199507000-00011 7564969

[B68] Tidball J. G. Villalta S. A. (2010). Regulatory Interactions between Muscle and the Immune System during Muscle Regeneration. Am. J. Physiology-Regulatory, Integr. Comp. Physiol. 298, R1173–R1187. 10.1152/ajpregu.00735.2009 PMC2867520 20219869

[B69] Toumi H. F'guyer S. Best T. M. (2006). The Role of Neutrophils in Injury and Repair Following Muscle Stretch. J. Anat. 208, 459–470. 10.1111/j.1469-7580.2006.00543.x 16637872 PMC2100203

[B70] Uezumi A. Fukada S.-i. Yamamoto N. Takeda S. i. Tsuchida K. (2010). Mesenchymal Progenitors Distinct from Satellite Cells Contribute to Ectopic Fat Cell Formation in Skeletal Muscle. Nat. Cel Biol. 12, 143–152. 10.1038/ncb2014 20081842

[B71] Verdijk L. B. Snijders T. Drost M. Delhaas T. Kadi F. Van Loon L. J. C. (2014). Satellite Cells in Human Skeletal Muscle; from Birth to Old Age. Age 36, 545–557. 10.1007/s11357-013-9583-2 24122288 PMC4039250

[B72] Vignola A. M. Chanez P. Chiappara G. Merendino A. Zinnanti E. Bousquet J. (1996). Release of Transforming Growth Factor-Beta (TGF-Beta) and Fibronectin by Alveolar Macrophages in Airway Diseases. Clin. Exp. Immunol. 106, 114–119. 10.1046/j.1365-2249.1996.d01-811.x 8870708 PMC2200550

[B73] Virgilio K. M. Martin K. S. Peirce S. M. Blemker S. S. (2018). Agent-based Model Illustrates the Role of the Microenvironment in Regeneration in Healthy and Mdx Skeletal Muscle. J. Appl. Physiol. 125, 1424-1439. japplphysiol. 10.1152/japplphysiol.00379.2018 30070607 PMC6295486

[B74] Von Walden F. Gantelius S. Liu C. Borgström H. Björk L. Gremark O. (2018). Muscle Contractures in Patients with Cerebral Palsy and Acquired Brain Injury Are Associated with Extracellular Matrix Expansion, Pro‐inflammatory Gene Expression, and Reduced rRNA Synthesis. Muscle Nerve 58, 277–285. 10.1002/mus.26130 29572878

[B75] von Walden F. Jalaleddini K. Evertsson B. Friberg J. Valero-Cuevas F. J. Pontén E. (2017). Forearm Flexor Muscles in Children with Cerebral Palsy Are Weak, Thin and Stiff. Front. Comput. Neurosci. 11, 1–8. 10.3389/fncom.2017.00030 28487645 PMC5403928

[B76] Wang Y. Wehling‐Henricks M. Samengo G. Tidball J. G. (2015). Increases of M2a Macrophages and Fibrosis in Aging Muscle Are Influenced by Bone Marrow Aging and Negatively Regulated by Muscle‐derived Nitric Oxide. Aging Cell 14, 678–688. 10.1111/acel.12350 26009878 PMC4531081

[B77] Wang Y. X. Rudnicki M. A. (2012). Satellite Cells, the Engines of Muscle Repair. Nat. Rev. Mol. Cel Biol. 13, 127–133. 10.1038/nrm3265 22186952

[B78] Webster M. T. Manor U. Lippincott-Schwartz J. Fan C.-M. (2016). Intravital Imaging Reveals Ghost Fibers as Architectural Units Guiding Myogenic Progenitors during Regeneration. Cell Stem Cell 18, 243–252. 10.1016/j.stem.2015.11.005 26686466 PMC4744135

[B79] Weiss J. A. Maker B. N. Govindjee S. (1996). Finite Element Implementation of Incompressible, Transversely Isotropic Hyperelasticity. Comp. Methods Appl. Mech. Eng. 135, 107–128. 10.1016/0045-7825(96)01035-3

[B80] Wilensky U. Rand W. (2015). An Introduction to Agent-Based Modeling. The MIT Press. Available at: https://mitpress.mit.edu/books/introduction-agent-based-modeling.

[B81] Wosczyna M. N. Rando T. A. (2018). A Muscle Stem Cell Support Group: Coordinated Cellular Responses in Muscle Regeneration. Dev. Cel 46, 135–143. 10.1016/j.devcel.2018.06.018 PMC6075730 30016618

[B82] Wynn T. A. Vannella K. M. (2016). Macrophages in Tissue Repair, Regeneration, and Fibrosis. Immunity 44, 450–462. 10.1016/j.immuni.2016.02.015 26982353 PMC4794754

[B83] Xue Q. Lu Y. Eisele M. R. Sulistijo E. S. Khan N. Fan R. (2015). Analysis of Single-Cell Cytokine Secretion Reveals a Role for Paracrine Signaling in Coordinating Macrophage Responses to TLR4 Stimulation. Sci. Signal. 8, ra59. 10.1126/scisignal.aaa2155 26082435 PMC5735825

[B84] Zammit P. Heslop L. Hudon V. Rosenblatt J. D. Tajbakhsh S. Buckingham M. E. (2002). Kinetics of Myoblast Proliferation Show that Resident Satellite Cells Are Competent to Fully Regenerate Skeletal Muscle Fibers. Exp. Cel Res. 281, 39–49. 10.1006/excr.2002.5653 12441128

[B85] Zanou N. Gailly P. (2013). Skeletal Muscle Hypertrophy and Regeneration: Interplay between the Myogenic Regulatory Factors (MRFs) and Insulin-like Growth Factors (IGFs) Pathways. Cell. Mol. Life Sci. 70, 4117–4130. 10.1007/s00018-013-1330-4 23552962 PMC11113627

