# Supplementary figures and images for: A Coupled Mechanobiological Model of Muscle Regeneration In Cerebral Palsy
Source: Front Bioeng Biotechnol. 2021 Aug 27;9:689714. doi: 10.3389/fbioe.2021.689714 (PMC8429491; doi:10.3389/fbioe.2021.689714)

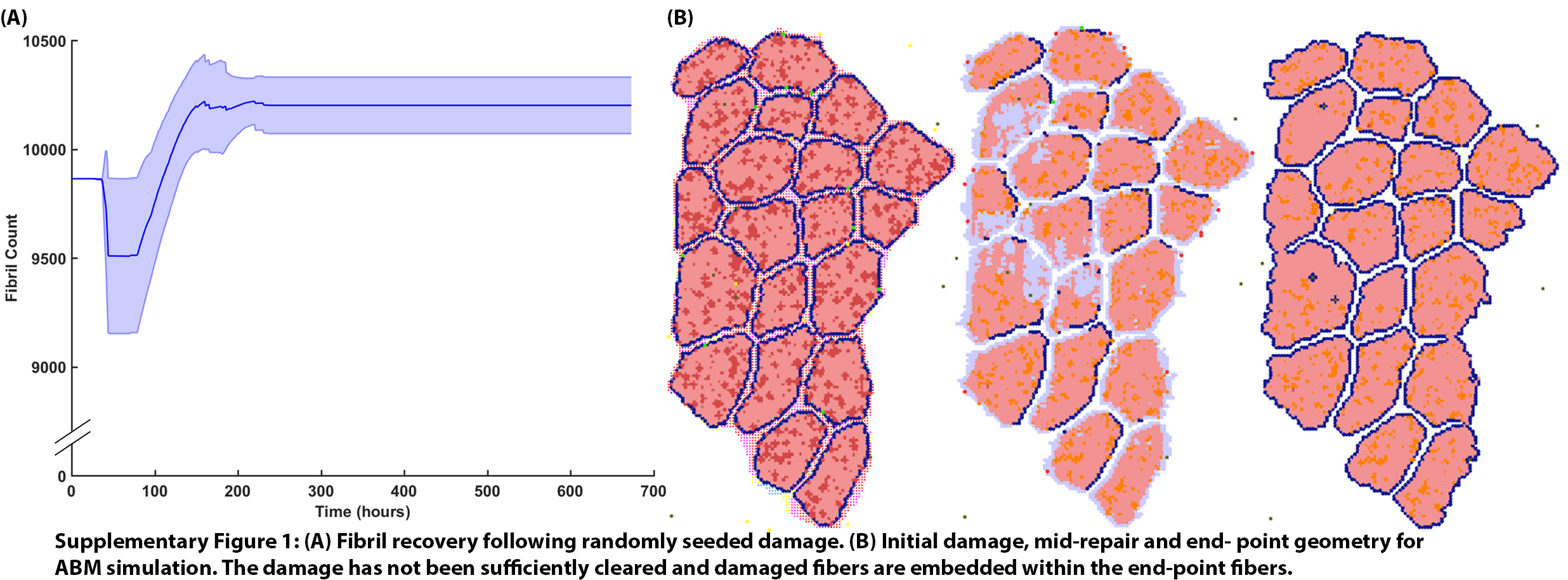

Supplement: Supplementary file 1 [file Image1.tif]
